# A scoping review of behavior change techniques used to promote physical activity among women in midlife

**DOI:** 10.3389/fpsyg.2022.855749

**Published:** 2022-09-21

**Authors:** Danielle Arigo, Kelly A. Romano, Kristen Pasko, Laura Travers, M. Cole Ainsworth, Daija A. Jackson, Megan M. Brown

**Affiliations:** ^1^Department of Psychology, Rowan University, Glassboro, NJ, United States; ^2^Department of Family Medicine, Rowan School of Osteopathic Medicine, Stratford, NJ, United States; ^3^Department of Psychology, Old Dominion University, Norfolk, VA, United States; ^4^Clinical Psychology Program, Chicago School of Professional Psychology, Washington, DC, United States

**Keywords:** women's health, midlife, physical activity, behavior change techniques (BCTs), behavioral intervention

## Abstract

Women in midlife experience health risks that could be mitigated by regular physical activity and reduced sedentary time, but this population rarely achieves physical activity levels that would protect their health. As a result, many behavioral interventions are designed to promote physical activity in this population, which are purportedly guided by theoretical models of health behavior (change) and activate an associated set of behavior change techniques (BCTs). The efficacy and effectiveness of these interventions appear to be limited, however, raising questions about their design and adaptation for women in midlife. Several aspects of these interventions are currently unclear. Specifically, which women they target (i.e., how “midlife” and “sedentary” or “inactive” are defined), which theoretical models or behavior BCTs are used, and how BCTs are activated in such interventions. A synthesis of this information would be useful as an initial step toward improving physical activity interventions for this at-risk group, and thus, represented the goal of the present scoping review. Eligibility required publication in a peer-reviewed journal in English between 2000 and 2021, inclusion of only women in midlife who did not have any medical or other restrictions on their physical activity (e.g., cancer diagnosis), and free-living physical activity or sedentary behavior as the target outcome (with associated assessment). Of the 4,410 initial results, 51 articles met inclusion criteria, and these described 36 unique interventions. More than half of the articles (59%) named an underlying theoretical model and interventions included an average of 3.76 identifiable BCTs (range 1–11). However, descriptions of many interventions were limited and did not provide enough detail to determine whether or how specific BCTs were activated. Interventions also used a wide range of inclusion criteria for age range and starting activity level, which has implications for targeting/tailoring and effectiveness, and many interventions focused on marginalized populations (e.g., women from racial/ethnic minority backgrounds, those un- or under-insured). The present review identifies some strengths and highlights important limitations of existing literature, as well as key opportunities for advancing the design and potential utility of physical activity interventions for women in midlife.

**Systematic review registration:**

https://osf.io/g8tuc.

## Introduction

Physical inactivity is a key contributor to the development of cardiovascular disease (CVD; Blair, 2009), which remains the leading cause of death worldwide. The arrival of menopause in midlife confers additional CVD risk for women (Matthews et al., 2009; Karvinen et al., 2019); this change coincides with a decrease in physical activity, which widens the gender disparity in physical activity engagement that favors men throughout the lifespan (Caspersen et al., 2000; Troiano et al., 2008). Consequently, women experience increased CVD risk during midlife, and physical activity is a key method for reducing the development and progression of CVD in this population. Among women, physical activity is protective against specific CVD risk conditions such as metabolic syndrome, type 2 diabetes, stroke, and coronary heart disease (Hu et al., 2000; Gill and Cooper, 2008; Lin et al., 2015) and also protects against cancer and osteopenia (Lynch et al., 2011; Moore et al., 2016; Watson et al., 2018).

A common approach to promoting physical activity is to deliver a behavioral intervention. This umbrella term is used to describe programs that employ individual or group discussions with a skilled facilitator to increase psychological skills that support physical activity engagement in daily life (e.g., using behavioral, cognitive, emotional, or social approaches). These interventions are delivered face-to-face, remotely (e.g., via phone), or using multiple modalities. Many researchers and clinicians suggest that women in midlife would benefit from behavioral interventions that are intentionally tailored to their unique needs. For example, in addition to the eventual onset of menopause and other physical changes, women in this developmental period experience changes in their caregiving and work demands, financial stability, and social networks that can affect their physical activity (Im et al., 2010). As a result, many physical activity interventions ostensibly address these needs and recruit and enroll only this population.

Yet, there appears to be no single definition of “midlife,” as experts acknowledge disagreement about the boundaries this period (Infurna et al., 2020). It may include any range between the ages of 30 and 69 years, and researchers may define “midlife” in a number of ways. Some studies appear to focus even more narrowly on subgroups of women in midlife, such as those from a particular racial or socioeconomic background (e.g., Koniak-Griffin et al., 2015). Despite their frequent participation in behavioral physical activity intervention trials open to adults (Waters et al., 2011; Cooke and Jones, 2017) and the preponderance of interventions that are described as tailored for their needs, however, women in midlife consistently show low physical activity engagement (Lee and Ory, 2013; National Center for Health Statistics, 2019). The characteristics of physical activity behavior change interventions that purportedly are tailored for this population warrant further investigation, to shed light on this discrepancy.

### Behavior change techniques in health behavior interventions

Behavioral or psychological theories are often used to guide the development of interventions to promote physical activity and other health behaviors. These theories suggest that specific experiences or constructs (e.g., motivation to engage in physical activity) are facilitators of or barriers to physical activity behavior; interventions then are designed to increase facilitating factors and decrease barriers by harnessing specific mechanisms of action associated with behavior change. The methods by which behavior change mechanisms are activated in interventions are called *behavior change techniques*, or BCTs (Abraham and Michie, 2008; Michie et al., 2011, 2013).

Notably, whereas BCTs provide insight regarding *how* interventions may promote adaptive health-related outcomes, theories provide a framework for understanding why health behaviors change post-treatment. For example, Social Cognitive Theory, which has commonly been used to guide physical activity promotion interventions among women (Perez et al., 2010; Jenkins et al., 2017; Flannery et al., 2019; Joseph et al., 2019), posits that learning and subsequent behavior change occurs as a function of reciprocal and dynamic interactions between individuals, their behavior, and their sociocultural environments (Bandura, 1998). A key component of this theory is that self-efficacy for changing physical activity (i.e., appraisal of one's own ability to make changes in this domain; Bandura, 1982) represents a powerful proximal determinant of behavior change. Consequently, interventions that are informed by this perspective frequently implement BCTs such as modeling the targeted behavior and reflecting on past successes. These BCTs are expected to account for the intrapersonal and contextual factors that are theorized to promote positive health outcomes (Gilinsky et al., 2015; Jenkins et al., 2017; Flannery et al., 2019) – specifically, increasing self-efficacy for engaging in physical activity as a mechanism to promote physical activity behavior change.

Although such theories frequently are used to inform the development of physical activity interventions and the selection of BCTs to be implemented, there is a considerable lack of consistency in the design and implementation of these treatments (Prestwich et al., 2014; Silva et al., 2014), as published descriptions of intervention trials do not always describe the specific features that activate BCTs. For example, several intervention descriptions indicate that participants self-monitor their physical activity behavior, but do not indicate how participants were taught to do this (e.g., using what methods, how often, and how any feedback offered should be used to inform physical activity decisions). Similarly, content often is delivered in groups “to facilitate social support,” but descriptions do not provide details about how the group was used to activate social support (e.g., the content of discussions, specific activities, communication between group meetings). In interventions that are described as tailored for populations such as women in midlife, additional information about the BCTs included and the intervention features that engage them would help to synthesize existing knowledge and suggest next steps for more effective physical activity promotion in these groups.

### Aims of the present review

Reviews and meta-analyses that have examined physical activity interventions among women have generally focused on intervention effectiveness (Perez et al., 2010; Jenkins et al., 2017; Flannery et al., 2019; Joseph et al., 2019), included women in a variety of developmental periods (e.g., only young and/or older adulthood, any age 18 or older; Perez et al., 2010; Anderson et al., 2014; Jenkins et al., 2017; Sherifali et al., 2017; Flannery et al., 2019; Joseph et al., 2019), or targeted women with specific experiences (e.g., pregnant or postpartum women; Gilinsky et al., 2015; Sherifali et al., 2017; Flannery et al., 2019). No existing review has focused on physical activity interventions that are described as tailored to the needs of women in midlife, the BCTs that are included in physical activity interventions for this group, or the theoretical rationale(s) guiding BCT selection.

To provide information specific to the design and content of physical activity interventions among women in midlife, the aims of the present review were to identify the definitions of midlife, the behavior change theories and associated BCTs that have been included in the design of physical activity programs for women in this age range, and any available details about their implementation. As an initial attempt to summarize this literature using broad research questions about intervention targets, we selected a scoping review approach, rather than a typical systematic review (which focuses on questions of intervention efficacy/effectiveness). Results may serve as an important initial step toward improving the understanding of how and why this population generally experiences modest outcomes following physical activity interventions and, in turn, guide recommendations to enhance intervention development.

## Methods

This review followed the initial guidelines delineated by Arksey and O'Malley (2005) and the recent PRISMA Extension for Scoping Reviews (PRISMA-ScR; Tricco et al., 2018). Research questions and methods were registered with the Open Science Framework prior to data extraction (https://osf.io/g8tuc). The original procedure indicated that articles published by September 30, 2020 would be included; the search was updated in July 2021 to include all eligible articles published by June 30, 2021.

### Research questions

For the present review, the following research questions were explored:

1) Which women have been the focus of physical activity programs designed for women in midlife?
2) Which theories have guided the design of physical activity programs designed for women in midlife?
3) Which behavior change techniques are used in physical activity programs designed for women in midlife?
4) How are these behavior change techniques implemented in physical activity programs designed for women in midlife?

### Article identification

Articles that met the following criteria were included in the present review: (1) available in English, (2) published between January 1, 2000 and June 30, 2021, (3) published in a peer-reviewed journal, (4) described the outcomes of a behavioral intervention to promote free-living physical activity or exercise, or reduce free-living sedentary time, (5) a primary behavioral target and outcome was either self-reported or objectively assessed physical activity, exercise, or sedentary time, (6) the target population was women in midlife, and (7) the target population did not pose any physical activity restrictions or guidelines (e.g., cancer, high risk of falling, etc.). Articles were excluded from the review if they failed to meet these criteria; if the intervention aimed to improve immediate physiological outcomes (walking/running speed, gait, VO_2_ max), rather than to promote free-living physical activity/exercise or reduce free-living sedentary time; or if physical activity, exercise, or sedentary time outcomes were not measured with quantitative metrics.

We elected to include only studies published since 2000 to ensure that conclusions reflect the recent state of the literature in this area, which include increasing appreciation for the unique physical activity needs of women in midlife (Im et al., 2010) and various delivery modes that were not commonly used before 2000 (e.g., web-supported remote delivery; Arigo et al., 2019). To provide the most comprehensive overview of physical activity intervention techniques for women in midlife, we also elected to include interventions that focused on physical activity as one primary component of an intervention, but included other targets (e.g., weight loss, dietary change, stress management). We detected no meaningful differences in the use of BCTs between interventions focused exclusively on physical activity and those that had other target health outcomes. Consequently, all studies included in this review are considered together.

**Figure 1:**
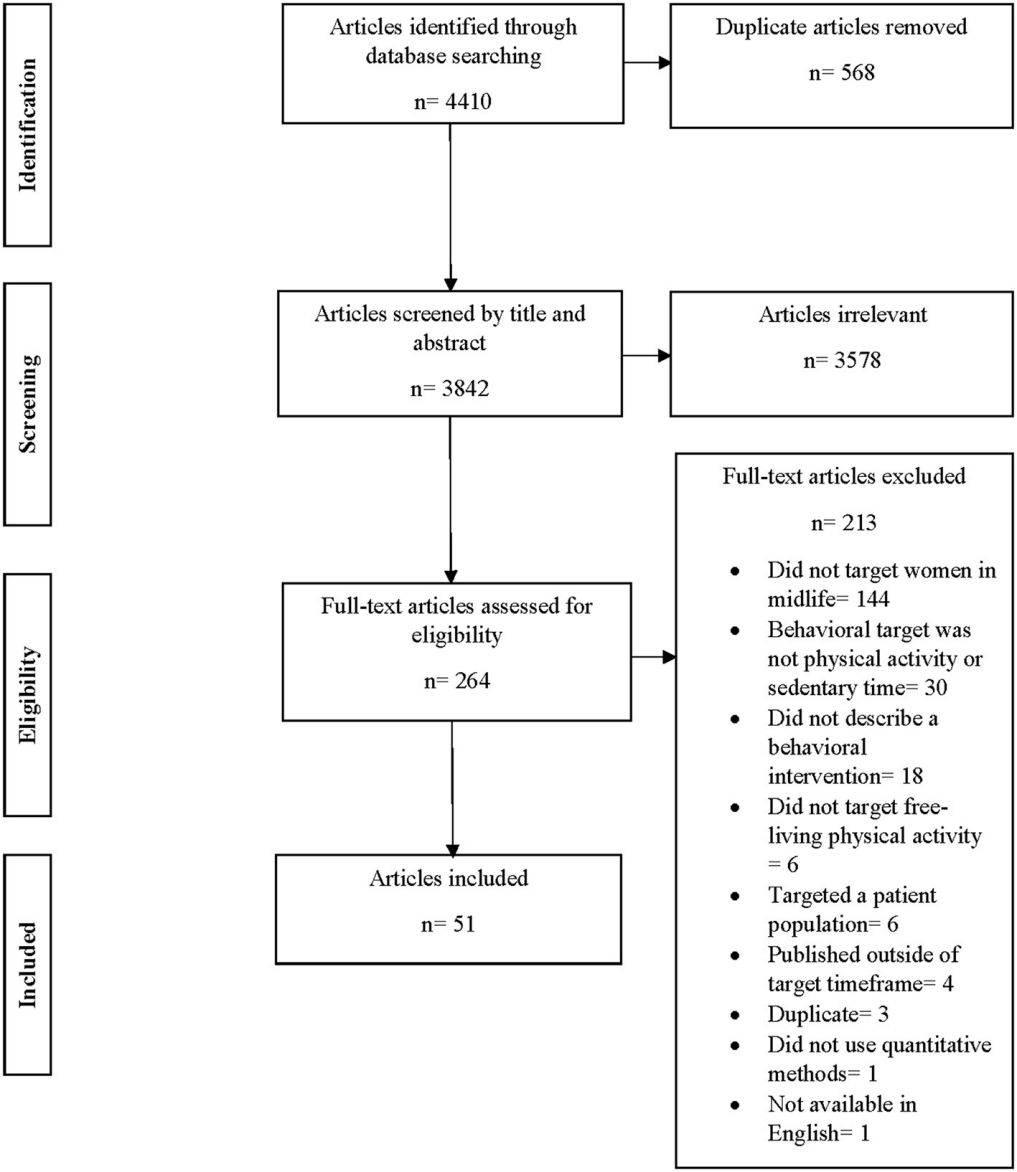
PRISMA flowchart of selection, sorting, and data extraction.

The authors searched for relevant articles on PsycInfo, PubMed, Web of Science, and CINAHL databases using the search terms “women,” “woman,” “female,” “midlife,” “middle age,” “middle adulthood,” “physical activity,” “exercise,” “sedentary behavior,” “active living,” “aerobic exercise,” “intervention,” “treatment,” “behavioral,” or “NOT cancer.” A total of 4,410 articles were returned from this search. After removal of 568 duplicates, 3,842 articles remained. Each article was evaluated by one author for this initial stage, to determine whether it met inclusion criteria based on screening the title and abstract. A total of 3,563 articles were excluded, leaving 264 articles for full text screening by six of the authors (KR, KP, LT, MCA, DJ, and MB). Two of these authors screened the full text of each article to ensure inter-rater agreement, and discrepancies were resolved by consensus with the first author (DA). During the full text screening, 213 articles were removed and 51 remained for the final stage of data extraction (see Figure 1).

### Data extraction

Data from the final set of 51 included articles were extracted by the authors. The following characteristics were extracted from each of the included articles: author(s) and year, journal published, sample size, sample characteristics, and definition of midlife (operationalized by inclusion criteria). Also extracted were data pertaining to the format and modality of intervention delivery, whether there was a control or comparison condition, and the reported theoretical grounding (if included).

Finally, BCTs and intervention features that were described as activating BCTs were extracted. An initial set of BCTs were selected for extraction based on the authors' familiarity with physical activity intervention research (specific to women in midlife and more broadly; Arigo, 2015; Butryn et al., 2016; Arigo et al., 2020a,b). These BCTs included the following [with Michie et al. (2013) associated numeric codes]: goal setting (proximal behavior [1.1] and/or distal outcome [1.3]), self-monitoring (proximal behavior [2.3] and/or distal outcome [2.4]), providing feedback (2.2), problem-solving and/or planning (1.4 and 1.2, respectively), social support (3.1–3.3), social comparison (6.2), and behavioral modeling (6.1). Additional BCTs described or alluded to in each article were also extracted, to provide a comprehensive overview of work in this area. These additional BCTs are grounded in behavioral and cognitive intervention traditions (e.g., stimulus control [12.3], reframing [13.2]). Each reviewer coded an average of 16 articles, with two reviewers per article. Comparisons between the two reviewers' coding of data extracted showed 80% agreement; discrepancies between reviewers were identified by DA and were resolved by a third coder.

## Results

The 51 articles included for review described 38 distinct physical activity interventions for women in midlife; 13 of these articles (25%) reported on secondary analyses from an existing intervention (see Supplementary material). Across primary and secondary outcome reports, 42 articles (82%) described randomized, controlled trials with comparison conditions, and 9 articles (28%) reported the results of single-arm pilot trials (including pre- to post-intervention change in physical activity and other outcomes). Sizes of samples included in analyses of intervention effects ranged from 18 to 645 women. Approximately equal proportions of articles indicated that the format of intervention delivery was in groups (*k* = 16, 31%), individual meetings (*k* =19, 37%), or a mix of both (*k* = 16, 31%). With respect to delivery modality, approximately half of included articles (*k* = 29, 57%) described delivery face-to-face/in-person only, and 3 (6%) described remote delivery (i.e., *via* mail or by telephone call). The remaining articles (*k* = 19, 37%) indicated delivery using a hybrid method (i.e., combination of face-to-face and remote modalities; see Supplementary material).

Across the articles included, “midlife” was defined in several different ways. This included use of various age ranges between 30 and 69 years old (e.g., 35–54, 40–65, 45–60; 49–69) and menopause status (e.g., postmenopause; see Supplementary material). Less than half (*k* = 18, 35%) indicated that eligibility required baseline physical activity to be below a certain threshold, and a total of 8 different thresholds were reported (e.g., < 150 mins of moderate-to-vigorous physical activity per week, no regular exercise). The majority of articles (*k* = 31, 60%) also indicated that they recruited a specific subset of women in midlife, such as those who work in school systems (e.g., Gebretatyos et al., 2020), women with overweight or obesity (e.g., Kuller et al., 2012), or underserved groups (e.g., those below the poverty line; Samuel-Hodge et al., 2009).

Notably, 35% of the included articles (*k* = 18) indicated that interventions targeted women in midlife with marginalized or multiply marginalized identities. For example, 18% (*k* = 9) of the included articles noted that their programs recruited women in midlife who were primarily un- or under-insured, or otherwise had low socioeconomic statuses (Keyserling et al., 2008; Samuel-Hodge et al., 2009, 2013; Hayashi et al., 2010; Khare et al., 2012; Scarinci et al., 2014; Agomo et al., 2015; Thomas et al., 2016; Kim et al., 2019). An additional 27% (*k* = 14) mainly or exclusively included women in midlife who identified with various racial or ethnic minority identities (Fitzgibbon et al., 2005; Gaston et al., 2007; Wilbur et al., 2008, 2016, 2017; Zenk et al., 2009; Sharpe et al., 2010; Khare et al., 2012; Samuel-Hodge et al., 2013; Scarinci et al., 2014; Agomo et al., 2015; Koniak-Griffin et al., 2015; Thomas et al., 2016; Gebretatyos et al., 2020).

Nearly all of these articles (33%, *k* =17) noted that their respective programs tailored their recruitment strategies and/or interventions to meet the needs of their targeted populations (Fitzgibbon et al., 2005; Gaston et al., 2007; Keyserling et al., 2008; Wilbur et al., 2008, 2016, 2017; Samuel-Hodge et al., 2009, 2013; Zenk et al., 2009; Hayashi et al., 2010; Sharpe et al., 2010; Khare et al., 2012; Scarinci et al., 2014; Agomo et al., 2015; Koniak-Griffin et al., 2015; Thomas et al., 2016; Kim et al., 2019). For example, to meet the needs of women in midlife with low socioeconomic status, many of whom identified as Hispanic (80%) and primarily spoke Spanish, the Be Wise program included intervention materials (e.g., handouts, a participant guide, etc.) that were written at a fourth-grade reading level in both English and Spanish, and included dietary interventions focused on foods that are common in the Hispanic culture and that could be purchased at grocery stores that accepted food stamps (Agomo et al., 2015). Likewise, in an intervention for women in midlife who identified as African American or Black and exhibited overweight or obese BMIs, culturally appropriate recruitment and intervention materials were developed to identify specific media (e.g., television and print materials), locations (e.g., churches), and core cultural values (e.g., promoting community cohesion, family/providing childcare, addressing religion/spiritual needs), to aptly recruit and engage this population (Fitzgibbon et al., 2005).

### Theoretical underpinnings

A total of 30 articles (59%) indicated that the intervention described was based on an identifiable theoretical model (see Supplementary material). The development of physical activity promotion interventions included in the present review was guided most often by Social Cognitive Theory (*k* = 13, 25% of all included articles). For example, one study developed intervention modules with goal setting (BCT codes 1.1 and 1.3) and identification of rewards (many possible BCT codes), in line with this theory's focus on self-efficacy, perceived barriers and motivators, and perceived risks and benefits (Agomo et al., 2015). Five additional papers described self-efficacy as promoted *via* group discussions, either as an explicit discussion topic (Shirazi et al., 2007; Wilbur et al., 2016; Kim and Kang, 2020) or enhancement by focusing the conversation on participants' successes (BCT code 15.3; Wilbur et al., 2005; Costanzo et al., 2006). Three articles referenced self-efficacy as a topic of take-home workbooks (Napolitano et al., 2006; Ehlers et al., 2015) or videos (Nazari et al., 2020) provided to each participant, and 1 indicated the use of verbal persuasion from intervention facilitators or researchers during face-to-face sessions (BCT code 15.1; Ortí and Donaghy, 2004). Additional articles described self-efficacy as promoted via BCTs such as goal setting (BCT codes 1.1 and 1.3) and social support (from group members and intervention facilitators; BCT codes 3.1–3.3; Anderson et al., 2006) or did not explain how self-efficacy was bolstered by the intervention (Gaston et al., 2007).

Another popular theory used to inform interventions was the Transtheoretical Model (*k* = 8, 16%). Aligned with this model, one study provided instructional sessions tailored to participants' stage of change, which included educational material, group discussions, and reflections on individuals' perception of susceptibility (BCT code unclear; Shirazi et al., 2007). Other theories used to guide intervention development included Cognitive Behavioral Theory (*k* = 4, 8% of all included articles); Interaction Model of Client Health Behavior (*k* = 3, 6%); Person, Extended Family, Neighborhood Model (PEN; *k* =2, 5%); Self-Efficacy Model (*k* = 1, 3%); Health Promotion Model (*k* =1, 3%); Revised Health Belief Model (*k* =1, 3%); and Social Marketing Framework (*k* =1, 3%). Multiple studies (*k* = 8, 16%) were described as integrating more than one theoretical or conceptual model to inform the design of their experimental interventions (see Supplementary material).

In line with several of these theories, 9 articles (17%) reported that interventions focused on increasing *motivation* for physical activity. Many did not describe the specific BCT intended to increase motivation. For example, five articles indicated that motivation was a topic of discussions in group, individual interactions, or workshops (Fitzgibbon et al., 2005; Zenk et al., 2009; Butryn et al., 2016; Wilbur et al., 2016; Kim and Kang, 2020); 4 articles referenced providing motivational messages outside of live interactions, via mail or email (Sharpe et al., 2010), telephone (Wilbur et al., 2017), text message (Kim et al., 2019), or videotape (Keyserling et al., 2008). None of these articles described or provided examples of the content of motivational messages, though one linked to an external, supplementary resource that did include examples (Sharpe et al., 2010).

Notably, several articles (*k* = 21, 41%) did not indicate *how* theories or conceptual models were used to inform either the design of their interventions or the selection of BCTs. Some articles that claimed that an intervention was informed by specific theory appeared to lack sufficient information about how their intervention was informed by that theory (Low et al., 2015) or contained intervention descriptions in separate protocol articles (Napolitano et al., 2006; see Availability of Additional Information, below).

### Goal setting (BCT codes 1.1 and 1.3)

Goal setting was reported as included in 49% of the articles meeting the present inclusion criteria (*k* = 25; see Supplementary material). Approximately half of these articles (*k* = 13) provided very brief descriptions that “goal setting” served as a topic of discussion during group sessions (Carels et al., 2004; Fitzgibbon et al., 2005; Gaston et al., 2007; Scarinci et al., 2014), individual consultations with study personnel (Anderson et al., 2006; Wilbur et al., 2008; Hayashi et al., 2010; Long et al., 2013; Koniak-Griffin et al., 2015; Low et al., 2015), or via print or web-based study materials (Ortí and Donaghy, 2004; Napolitano et al., 2006; Sharpe et al., 2010), but did not provide specifics on the nature of goal-setting discussions or materials. In contrast, 12 articles provided more specific details on how goal setting was incorporated (Peterson et al., 2005; Costanzo et al., 2006; Keyserling et al., 2008; Samuel-Hodge et al., 2009, 2013; Agomo et al., 2015; Butryn et al., 2016; Wilbur et al., 2016, 2017; McGuire et al., 2019; Nazari et al., 2020; Shariati et al., 2021), and there was considerable variability in how goal setting was implemented across these articles.

For example, 3 articles described helping participants learn how to set specific behavioral goals that had clear parameters (BCT code 1.1). Two involved individual consultations with study personnel who provided support for developing SMART physical activity goals (Specific, Measurable, Achievable, Relevant, and Time-bound; Costanzo et al., 2006; McGuire et al., 2019). The third article indicated that participants were led to set 2–3 physical activity goals by their intervention facilitators during individual sessions, with follow-up and goal re-setting as these sessions progressed (Keyserling et al., 2008). An additional 2 articles used a multi-faceted approach to goal setting, wherein participants were asked to set and reset goals using more than one format or modality. For example, 1 article indicated that goal setting occurred in both group discussions and individual telephone calls with health advisors between group sessions (Keyserling et al., 2008). The other described goal-setting discussions in face-to-face groups as well as via digital group message boards on the Fitbit platform (Butryn et al., 2016).

### Self-monitoring (BCT codes 2.3 and 2.4)

Self-monitoring was incorporated in 26 articles (51%), using a variety of methods (see Supplementary material). For example, 19 articles indicated that participants tracked their physical activity behavior (BCT code 2.3) in paper and pencil logs, with data from pedometers (Keyserling et al., 2008; Sharpe et al., 2010; Ribeiro et al., 2014; Conroy et al., 2015; Koniak-Griffin et al., 2015), accelerometers (Carels et al., 2004), physiological data (e.g., heart rate, rate of perceived exertion during physical activity; Ortí and Donaghy, 2004; Asbury et al., 2006), and/or self-reports (e.g., total minutes of physical activity per day; Wilbur et al., 2001, 2005, 2008; Simkin-Silverman et al., 2003; Carels et al., 2004; Anderson et al., 2006; Costanzo et al., 2006; Ludman et al., 2009; Samuel-Hodge et al., 2009, 2013; Zenk et al., 2009; Sharpe et al., 2010; Hollis et al., 2015).

Similarly, 5 articles described logging physical activity behavior (BCT code 2.3) in web-based platforms, based on data from pedometers (Ehlers et al., 2015), physiological measures (e.g., heart rate during physical activity from heart rate monitors; Wilbur et al., 2001, 2005, 2008), or self-reports (Cussler et al., 2008). One article indicated that participants used commercially available wearable sensors (i.e., Fitbit) to self-monitor their physical activity, and their physical activity totals were available in real time via the device and associated web and mobile platforms (Butryn et al., 2016). In other studies (*k* = 3), data from pedometers (Kim et al., 2019) or accelerometers (Wilbur et al., 2016, 2017) were regularly reported by participants to (automated) telephone response systems for self-monitoring purposes. A final article noted that self-monitoring was discussed in group sessions (Fitzgibbon et al., 2005), without specifying what these discussions entailed or which self-monitoring method was used.

### Providing feedback (BCT code 2.2)

Fifteen articles (29%) described interventions that provided participants with feedback on their engagement in physical activity. Most of these included only brief descriptions indicating that feedback was provided by research staff or intervention leaders (*k* = 9; Simkin-Silverman et al., 2003; Peterson et al., 2005; Wilbur et al., 2005; Shirazi et al., 2007; Keyserling et al., 2008; Samuel-Hodge et al., 2009; Scarinci et al., 2014; Butryn et al., 2016; Mirzaei et al., 2020) and/or web-based automatic response systems (*k* = 4; Wilbur et al., 2005; Cussler et al., 2008; Ehlers et al., 2015; Butryn et al., 2016). This BCT was described as providing participants with feedback on their current level of physical activity or the extent to which they attained their physical activity goals, without additional detail (including how often participants received feedback).

As exceptions, 4 articles provided more specific information on how and what types of feedback was provided during their interventions. In a print-based physical activity promotion intervention, participants were asked to complete a comprehensive questionnaire at 4 points during the intervention (i.e., at baseline and months 1, 3, and 6; Napolitano et al., 2006). Data from these questionnaires were used to generate individually tailored feedback reports that were mailed to participants, and targeted factors such as self-efficacy, barriers, benefits, social support, and goal setting related to physical activity. In addition, participants in a 4-session intervention were provided with feedback on their physiological responses to a supervised physical activity session (i.e., achieving between 60–85% of their maximal heart rates during physical activity, rate of perceived exertion; Ortí and Donaghy, 2004).

Other articles reported that their interventions provided feedback via both research staff/intervention leaders and use of reports generated from automatic response systems. For example, participants received supportive feedback tailored to their current stage of change for increasing physical activity, compiled from self-monitoring data that participants entered into an automated telephone system and delivered by study personnel (Wilbur et al., 2008). Similarly, phone calls were used to provide feedback from an interventionist regarding step counts that participants reported to an automated telephone system (Wilbur et al., 2016). This feedback incorporated use of motivational interviewing techniques and was only provided after the interventionist and participant discussed the latter's progress in attaining their physical activity goals.

### Problem-solving and planning (BCT codes 1.4 and 1.2)

Eleven articles (22%) indicated that problem-solving skills were taught in the intervention (BCT code 1.4); 10 used group or individual discussion time to cover these topics (Wilbur et al., 2001, 2005, 2008; Keyserling et al., 2008; Ludman et al., 2009; Samuel-Hodge et al., 2009; Long et al., 2013; Ribeiro et al., 2014; Koniak-Griffin et al., 2015; Low et al., 2015), whereas 3 used print or emailed materials (alone or in conjunction with discussions; Wilbur et al., 2008; Sharpe et al., 2010; Long et al., 2013). The specific problem-solving skills were not described, with the exception of limited details provided by Scarinci et al. (2014) (i.e., problem-solving “steps” were taught and problem-solving “ability” was evaluated). Similarly, 11 articles referenced the use of planning and/or intention formation with respect to physical activity behavior (BCT code 1.2), using group or individual intervention time to set a weekly schedule for activity (date/time/location; Fitzgibbon et al., 2005; Wilbur et al., 2005; Anderson et al., 2006; Costanzo et al., 2006; Keyserling et al., 2008; Zenk et al., 2009; Agomo et al., 2015; Ehlers et al., 2015; Butryn et al., 2016; McGuire et al., 2019) or being guided through the process with a video (Nazari et al., 2020).

### Social support (BCT codes 3.1–3.3)

Social support was reported as an included BCT in 23 studies (45%; see Supplementary material). Of these, 17 studies provided opportunities for or reviewed tips on obtaining support in person or in online group activities. To achieve this, 10 of the articles that referenced social support used group sessions, 3 articles used partner/group walks, and 2 used online community message boards. For example, one article described a focus on participants eliciting social support from family and friends by engaging in role plays during group discussion (Costanzo et al., 2006). Of the articles that described incorporating social support, 10 facilitated support via individual tailored sessions, psychoeducational resources, and materials on eliciting support for participants or their supporters to utilize independently (i.e., e-book, pamphlet, intervention staff feedback). For instance, multiple studies used individual coaching via phone or in-person sessions from study staff tailored to participant needs (e.g., Wilbur et al., 2005; Zenk et al., 2009; Koniak-Griffin et al., 2015). Articles that referenced social support as a BCT were approximately evenly split between those facilitating support from intervention leaders and members (*k* = 9, 52%), and those providing discussion and tips for participants to elicit support from family and friends (*k* = 8, 48%). Importantly, several studies claimed to use social support but either did not meet BCT criteria (i.e., “prompting the person to plan how to elicit support from other people to help him/her achieve their target behavior/outcome;” Michie et al., 2011) or provided insufficient detail to discern whether criteria were met, and many descriptions lacked information about how social support was facilitated.

### Social comparison (BCT code 6.2)

Only 2 articles (5%) explicitly referenced social comparisons, or self-evaluations relative to others (Festinger, 1954). Peterson et al. (2005) indicated that their intervention was based on an extension of Social Comparison Theory, which identified comparison as underlying the effects of social support (Wills, 1985). However, the BCTs described were distinct aspects of social support (e.g., instrumental vs. emotional support; BCT codes 3.2 and 3.3, respectively), activated via group discussions and workbook activities. In contrast, Butryn et al. (2016) differentiated social comparison processes from those of social support, and engaged comparison as a BCT via data sharing. Specifically, this intervention used wearable physical activity monitors connected to a digital platform and an associated leaderboard, which ranked participants' physical activity behavior (e.g., steps per day) from most to least. All participants had access to this feature, which updated physical activity rankings in real time to facilitate physical activity-based social comparisons. One additional article (Nazari et al., 2020) indicated the use of a video testimonial from a woman in midlife who had lost 17 kg of her weight by increasing physical activity, though this was not specified as intended to activate social comparison processes.

### Behavioral modeling (BCT code 6.1)

Thirteen articles (25%) described the use of behavioral modeling as a BCT. Modeling was provided through live demonstrations of activities during group sessions (e.g., Fitzgibbon et al., 2005; Keyserling et al., 2008; Samuel-Hodge et al., 2009, 2013; Koniak-Griffin et al., 2015), via video demonstrations of activities (e.g., Wilbur et al., 2008, 2016; Nazari et al., 2020), or both (e.g., Costanzo et al., 2006). Another approach reported was to have facilitators hand out photographic illustrations of activities during intervention sessions (McGuire et al., 2019). Models were intervention facilitators, peers who were already successful with physical activity, or professionals (e.g., athletes, coaches). Though in many cases, the model was not specified (e.g., Gaston et al., 2007; Wilbur et al., 2017).

### Other BCTs: Skills rooted in behavioral and/or cognitive therapy

Additional BCTs described in classic behavioral terms included stimulus control (BCT code 12.3; *k* = 3; Fitzgibbon et al., 2005; Shirazi et al., 2007; Butryn et al., 2016) and reinforcement/reward management (BCT codes 10.2, 10.3, 10.4, 10.9, and 14.10; *k* = 4; see Supplementary material; Ortí and Donaghy, 2004; Fitzgibbon et al., 2005; Shirazi et al., 2007; Agomo et al., 2015). These were either topics of intervention discussions or included as content in take-home workbooks. Of note, 4 articles indicated that incentives (BCT code 10.1) were used by program staff to reinforce goal attainment and enhance self-esteem (e.g., Peterson et al., 2005; Agomo et al., 2015). For example, participants in 2 interventions could earn items such as food storage containers, aprons, exercise videos, cookbooks, stick blenders, and pedometers for meeting their weekly physical activity goals (Samuel-Hodge et al., 2009, 2013). Other cognitively oriented BCTs that appeared in intervention descriptions included “self-evaluation” and setting realistic expectations (possible BCT code 15.4; Wilbur et al., 2016), cognitive restructuring (possible BCT code 13.2; Carels et al., 2004), and “cognitive-behavioral strategies” (unspecified; Thomas et al., 2016).

### Summary and reported availability of additional information

Across all articles, the average number of identifiable BCTs reported as included in each intervention was 3.76 (*SD* = 2.51), with a range of 1 to 11. Of note, 20 articles referenced outside sources as containing relevant details about intervention BCTs and their implementation (i.e., sources not included in the present review). Specifically, 10 articles referenced a protocol paper (1 that was under review at the time and appears to be unpublished), 2 referenced a website containing the intervention manual, and 9 referenced a previously published empirical paper that did not meet criteria for inclusion in this review. Thus, accessing critical details about the design and delivery of physical activity interventions for women in midlife required searching elsewhere for 40% of the articles included in this review.

## Discussion

The past 20 years have seen considerable interest in using behavioral interventions to promote physical activity among women in midlife, with emphasis on physical activity as a method of primary or secondary prevention of health problems associated with conditions such as cardiovascular disease, cancer, and osteopenia. Specifically, the present review identified 51 articles published between 2000 and 2021 that report on findings from such interventions, which describe 38 unique intervention programs. The majority of these programs are delivered face-to-face and use a group format, though subsets incorporate intervention delivery via telephone and individual discussions with treatment staff. Very few that primarily use live interactions with professional facilitators have added components delivered via mobile technology such as smartphones or tablets, and only 2 incorporated internet discussion boards to extend intervention contact between group sessions (Ehlers et al., 2015; Butryn et al., 2016). This represents a particular area of opportunity for future intervention adaptations, as digital components are both feasible and acceptable among women in midlife (Im et al., 2012; Joseph et al., 2021) and can extend content from intervention sessions to daily life.

One notable strength of existing research in this area is the representation of multiply marginalized women in midlife, such as those who identified as Black/African American, Hispanic/Latina, exhibited low socioeconomic status, were un- or under-insured, had higher body weights, and/or resided in rural areas (e.g., Fitzgibbon et al., 2005; Gaston et al., 2007; Keyserling et al., 2008; Hayashi et al., 2010; Khare et al., 2012; Agomo et al., 2015; Koniak-Griffin et al., 2015). Many studies also tailored their recruitment procedures (e.g., held meetings at local and easily accessible community centers) and intervention components (e.g., intervention leaders who were part of participants' local communities, ensured that written handouts contained information at a reading level that was appropriate for study participants; e.g., Gaston et al., 2007; Samuel-Hodge et al., 2009; Koniak-Griffin et al., 2015; Thomas et al., 2016) to meet the needs of these populations. Such efforts are valuable for engaging subgroups of women in midlife who may be at risk for suboptimal intervention engagement, as well as at risk for insufficient physical activity and poor health outcomes related to their multiply marginalized statuses (Tovar et al., 2018; White et al., 2020). These programs warrant continued evaluation and implementation to address persistent health disparities.

### BCTs included in physical activity interventions for women in midlife

In line with taxonomies described by Abraham and Michie (2008) and Michie et al. (2011, 2013), descriptions of the physical activity interventions that were included in the present review indicated intervention BCTs that targeted a variety of mechanisms of action. Most popular among these (included in approximately 50% of the identified articles) were self-monitoring and goal setting. Goal setting appeared to include both proximal behavior and distal outcomes (BCT codes, 1.3 and 1.1, respectively), though the former was much more common; self-monitoring appeared to focus on behavior (2.1), rather than outcomes (2.2), though this was not always clear from the description. Both self-monitoring and goal setting have been associated with positive changes in physical activity and related behavioral health outcomes across populations (Epton et al., 2017; Berry et al., 2021). Notably, these BCTs were described with some detail in several articles (e.g., described how and the methods that were used to incorporate and activate these BCTs), which is useful for other researchers to maintain consistency, determine replicability, and advance science in this area.

In contrast, although promoting social support was also well-represented in included articles (referenced in approximately 50%), there were few details regarding how this BCT was activated (BCT code 3.1, general). Thus, it is not clear which support types or associated BCT codes were included (i.e., practical vs. emotional support; 3.2 and 3.3, respectively). It is possible that the interventions described without sufficient detail expected that using a group format would automatically activate social support. Importantly, however, it is clear from BCT guidelines that activating social support requires more than simply bringing individuals together in groups; social support must be actively facilitated by the intervention process and/or content (Michie et al., 2013). This oversight may represent a critical limitation of existing physical activity interventions designed for women in midlife, and could be addressed with additional attention to facilitating social support in these programs. It is difficult to draw strong conclusions in this vein without additional information. Effectively activating social support may be particularly relevant and powerful for women in midlife, given the cognitive, physiological, and social changes and stressors that women commonly experience during this period of development (Infurna et al., 2020) and their demonstrated utility in other populations of women (e.g., Perez et al., 2010; Jenkins et al., 2017; Flannery et al., 2019; Joseph et al., 2019).

Critically, however, there was limited evidence to suggest that any of the BCTs included in physical activity interventions were tailored specifically for use with women in midlife. This is surprising, given the considerable existing evidence that tailored interventions and intervention components are associated with more favorable physical activity and related behavioral health outcomes than those that are not (Krebs et al., 2010; Berry et al., 2021). Lack of tailoring of behavioral BCTs in general, and self-monitoring and goal setting skills in particular, in physical activity promotion interventions for women in midlife may be a missed opportunity that could be addressed in future research. For example, interventions might specifically address how to set goals, effectively self-monitor, and recruit social support resources while managing work, caregiving, and physical changes such as those that accompany midlife and menopause. Tailoring BCTs for use with women in midlife may increase the perceived acceptability of physical activity promotion interventions for this population and prove helpful in addressing their low physical activity engagement rates (Lee and Ory, 2013; National Center for Health Statistics, 2019). Here too, however, it is difficult to make strong recommendations without additional information about the activation of these BCTs.

### Reporting limitations associated with existing intervention studies

Overall, the present review shows that only a few BCTs that are prevalent in physical activity interventions for women in midlife are described in detail, whereas many are referenced with little or no information about their activation. Further, the selected theoretical grounding and associated BCTs (e.g., those that support self-efficacy enhancement through the lens of Social Cognitive Theory) often are described as uniquely matched to the needs of women in midlife. This is particularly true for the inclusion of social support. Indeed, some research has documented lack of support as a barrier to physical activity engagement among women in midlife (e.g., Im et al., 2012), though this (or similar) evidence was not invoked to justify emphasis on activating social support to promote physical activity in this population. In general, very few articles included in this review clearly articulated the alignment between the specific needs of women in midlife, the theories or BCTs selected, and the processes used to activate the particular BCTs in interventions.

One view is that not providing this level of detail is justified, on multiple fronts. First, the primary goal of the articles included in this review was to report on intervention outcomes, and space restrictions in many journals prevent the inclusion of details about both intervention delivery and evaluation. The recent trend of publishing protocol papers may help to address this problem, especially given that many protocols are published with open access. However, this separates relevant details about intervention delivery from indicators of efficacy or effectiveness, both in terms of physical publication space and in terms of timing (as protocol papers often are published much earlier than outcomes). Thus, it can take considerable added effort to get a sense of which BCTs are used, with what rationale, and to what effect, slowing down progress toward effective physical activity promotion for women in midlife.

Second, detailed information about intervention delivery often is considered proprietary until (or long after) final-stage dissemination trials are complete, and may never become widely available. It may be prudent to avoid publicizing the details of an intervention until it has undergone thorough testing. As most publications tout the positive outcomes of each intervention, however, it would be useful for researchers to have more information about what, specifically, led to these outcomes. This is consistent with conclusions from prior meta-analyses, which have indicated lack of explicit identification (and measurement) of theoretically informed BCTs that are targeted by physical activity interventions, as well as a lack of information on how intervention content specifically maps onto the chosen theory's purported mechanisms of action (Prestwich et al., 2014). This lack of detail may be particularly problematic for populations such as women in midlife, for whom existing physical activity interventions have generally exhibited limited efficacy (Murray et al., 2017).

As a result, even with acknowledging these realities of publishing behavioral intervention research, overarching impressions of the state of this literature are that it offers little clarity or consensus about how to build effective physical activity interventions for women in midlife, and that there have been few innovations in this field over the past 20 years. With respect to building interventions, the programs were described in too little detail to be replicated and extended in future work by other clinical researchers. Without an understanding of how these interventions activate the intended BCTs, researchers intending to advance the field have to rely on other information, such as their own previous experience (which may be idiosyncratic or inaccurate with respect to existing work). This is particularly problematic for interventions shown to be superior to a comparison condition in large-scale trials (e.g., Khare et al., 2012; Scarinci et al., 2014), as these should be prime candidates for ongoing refinement and dissemination. Available literature thus offers little fodder for hypotheses about why certain physical activity interventions may be more effective for women in midlife than others or which BCTs should be retained; this may be one reason for repetition of several BCTs and combinations, rather than expansion with novel approaches.

### BCTs that warrant additional attention in future work with women in midlife

Several theories and associated BCTs are becoming more common in behavioral interventions to promote health behavior change, but have rarely been included in physical activity interventions for women in midlife. For example, mindfulness- and acceptance-based approaches to intervention teach participants to increase present-moment awareness of decision-making, connect their actions to their overarching life values, and tolerate temporary physical or emotional discomfort if actions align with their values (Hayes et al., 2006). In the context of physical activity intervention, physical activity engagement is presented as supportive of participants' values (such as overall health or caregiving for others); any efforts to focus on deliberate decision-making about physical activity and discomfort during physical activity (including boredom or dislike) are considered tolerable in the pursuit of a values-driven life (cf. Forman and Butryn, 2015). This perspective invokes BCTs such as reframing (BCT code 13.2), self-talk (15.4), and regulating negative emotions (11.2), albeit with specific techniques such as cognitive defusion (i.e., acknowledging thoughts and emotions without acting on them). Interventions that focus on promoting these skills have led to meaningful increases in physical activity among college students (Butryn et al., 2011) and adults with overweight and obesity (Butryn et al., 2018, 2021). Yet, mindfulness- and acceptance-based skills were not highlighted (or not described in sufficient detail) in studies included in this review.

Similarly, emerging evidence shows that activating social comparisons of physical activity in the context of digitally supported behavioral interventions is associated with physical activity increases among women (BCT code 6.2; Arigo, 2015; Arigo et al., 2015). In these interventions, participants have access to each other's real-time physical activity engagement (i.e., totals of steps and minutes of moderate- and vigorous-intensity activity), collected via wearable physical activity monitor; totals are ranked from most to least on a leaderboard to induce comparisons between participants. Women in midlife may be particularly responsive to social comparison opportunities, as their daily physical activity behavior changes with fluctuations in naturally occurring comparison activity (Arigo et al., 2021) and they identify lack of physical activity role models as a barrier to engagement (Vrazel et al., 2008). However, social comparison was activated in only one of the articles included in the present review (Butryn et al., 2016). Although a second article (Peterson et al., 2005) described its intervention as based on Social Comparison Theory (Festinger, 1954), the translation of this theory to the intervention components was not clearly delineated.

In contrast, behavioral modeling was referenced in 14 articles (27%; BCT code 6.1), whereby participants were able to view someone else performing a skill correctly (e.g., an intervention facilitator). Behavioral modeling may involve comparing oneself to the model to limit discrepancies between one's own and the model's behavior; however, modeling often involves comparing to someone perceived as an expert, whereas social comparison focuses on comparing to non-expert peers (e.g., other intervention participants; Abraham and Michie, 2008). It is not clear whether one is more effective for promoting physical activity among women in midlife, either overall or in certain intervention contexts. Thus, the use of BCTs based on promoting social comparison, mindfulness, and acceptance-based skills, as well as those that incorporate digital supports that can reach women in daily life, represent opportunities for introducing new techniques to physical activity interventions for women in midlife.

### Implications for promoting physical activity among women in midlife

The present review also illustrates the acknowledged lack of consensus about how to define “midlife,” as well as how to define “physically inactive.” Ages that qualified as midlife ranged from 30 to 69, and some studies used menopause status in lieu of an age range (e.g., Asbury et al., 2006; Gabriel et al., 2011; Hollis et al., 2014). Across studies, there also were 8 different thresholds used to indicate physical inactivity. These inconsistencies have been noted in recent work examining midlife health and sedentary lifestyles (Tremblay et al., 2017; Infurna et al., 2020), which raises critical questions about tailored physical activity promotion for women in this life stage: for whom are these interventions currently designed, and could increased consistency in the definitions of key criteria improve intervention development (as well as uptake, efficacy, and effectiveness)?

For instance, women at the ages of 65–69 may have very different physical and psychological needs than women at the ages of 30–35 (Berkman and Soh, 2017), and barriers to behavior change may look very different for someone already achieving 60 min of moderate-to-vigorous physical activity per week, relative to someone achieving only 10 (or 0; Powell et al., 2011). Attempting to address these wide ranges of needs in a single intervention program (relative to focusing on a smaller range of needs in more depth) may dilute its efficacy. Further, as many physical activity interventions for this population purport to foster social support between women as a BCT, heterogeneity between women may be counterproductive. Exposure to differing experiences can offer new perspectives, particularly if someone who has been through an experience can provide comfort or advice (cf. Kulik et al., 1996). However, support may be sub-optimally effective if women do not see group members as relatable or able to understand their challenges, or may be less beneficial for older women who share their experience (as there may be few opportunities for reciprocation; Carmack Taylor et al., 2007). As noted, greater attention to social support in physical activity interventions for women in midlife could be important for improving their efficacy and effectiveness (see Table 1). Overall, it appears that there is little consistency in the population of interest, which could hinder efforts to address physical inactivity in this group.

**Table 1:** Summary of key takeaways from the present review and recommendations for future research on physical activity interventions for women in midlife.

**Observation from literature synthesis**	**Recommendation for future work**
Use of a wide range of definitions for “midlife” and “physically inactive/sedentary.”	Examine heterogeneity in these areas as moderators of efficacy/effectiveness; include clear rationales for the definitions and criteria used in each study; test for the benefits of increasing heterogeneity within studies to maximize the potential power of BCTs such as social support and social comparison. Fieldwide, consider achieving greater consensus about these terms.
Predominant use of face-to-face intervention only (with some telephone support).	Explore the adjunctive use of digital components (e.g., online message boards) that can extend participant contact with intervention material and/or each other (e.g., for social support).
Many interventions designed for/included only women from marginalized groups (e.g., Black/African American women, women who were uninsured).	Continue to focus on promoting physical activity in marginalized groups using interventions adapted for their needs.
Very little detail provided regarding the activation of BCTs; not always clear which specific BCT was activated or how this would be replicated in ongoing intervention work.	Include detailed information about the specific BCTs included in the intervention and how these BCTs are activated (e.g., how specific types of social support were facilitated between group members); if space is limited, consider the use of Supplementary material.
Little evidence that the theoretical underpinnings or BCTs were selected for their relevance to women in midlife, or that BCTs were activated in a way that was tailored to the specific needs of this population.	Specify the population-specific rationale for a theoretical model and BCTs, and increase the use of BCTs/features that activate BCTs in ways that are tailored to the needs of women in midlife.
Very little engagement of social comparison processes or use of third-wave behavioral approaches to intervention, despite increasing evidence for their efficacy.	Include and test the contributions of social comparison features and techniques informed by mindfulness- and acceptance-based theories of health behavior change.

### Strengths, limitations, and additional future directions

To our knowledge, this is the first systematic overview of existing literature on behavioral interventions to increase physical activity among women in midlife. Given that this is an area of considerable interest (as evidenced by the number of relevant publications in the past two decades), the present review should be useful for providing researchers with a synthesis of existing approaches to intervention. This review also used pre-registered methods and relied on consensus across multiple coders to extract relevant information from each article. As we conducted a scoping review, rather than a traditional systematic review or quantitative meta-analysis, we are not able to draw conclusions about the efficacy of existing interventions or the BCTs that are most strongly associated with physical activity increases in this population. This is an important next step for research in this area and will help to advance the field, by identifying the BCTs that should be included in future interventions and those that may be eliminated or modified. This work would be most useful with the inclusion of age range (or other definition of midlife) and physical activity inclusion criteria as potential moderators, to determine whether greater participant homogeneity in interventions is associated with better outcomes.

Finally, it is critical that researchers understand not just which BCTs are associated with physical activity increases, but *how* these BCTs are activated to achieve these outcomes and how they are received by women in midlife. If protocol papers provide a useful outlet for presenting this information, publishing such papers should be an expected step in intervention development and testing. This likely will require ongoing financial resources to support publication (i.e., for open-access article processing costs), and researchers should plan to identify these types of papers in their preparation for intervention work, in addition to identifying papers that report on outcomes. An alternative to publishing a separate protocol paper would be to include more detailed information about an intervention in supplements for outcomes papers; supplemental sections now available for many publications and often appear online only. As there are several potential issues with the current use of supplemental sections, however (see Pop and Salzberg, 2015), these should be carefully considered and crafted to provide the most relevant information about the intervention. These steps also will help to advance the field by both increasing replicability and limiting time spent merely “reinventing the wheel” of physical activity interventions for women in midlife.

## Data availability statement

The original contributions presented in the study are included in the article/Supplementary material, further inquiries can be directed to the corresponding author/s.

## Author contributions

DA conceptualized the review, resolved discrepancies between coders, and supervised the project. KR, KP, LT, MA, DJ, and MB searched, sorted, and extracted data from articles. All authors contributed to the article and approved the submitted version.

## Funding

This work was supported by the National Heart, Lung, and Blood Institute (U.S. National Institutes of Health) under Grant K23HL136657 (PI: DA). The support time for KR was provided by National Institute of Mental Health (U.S. National Institutes of Health) under Grant F31MH120982 (PI: KR).

## Conflict of interest

The authors declare that the research was conducted in the absence of any commercial or financial relationships that could be construed as a potential conflict of interest.

## Publisher's note

All claims expressed in this article are solely those of the authors and do not necessarily represent those of their affiliated organizations, or those of the publisher, the editors and the reviewers. Any product that may be evaluated in this article, or claim that may be made by its manufacturer, is not guaranteed or endorsed by the publisher.

## References

[B1] Abraham C. Michie S. (2008). A taxonomy of behavior change techniques used in interventions. Health Psychol. 27, 379–387. 10.1037/0278-6133.27.3.379 18624603

[B2] Agomo H. C. Andresen P. A. Deshmukh D. (2015). Be wise: Implementing a lifestyle intervention to reduce stroke risk in low-income midlife women. J. Neurosci. Nurs. 47, 36–43. 10.1097/JNN.0000000000000108 25565593

[B3] Anderson D. Mizzari K. Kain V. Webster J. (2006). The effects of a multimodal intervention trial to promote lifestyle factors associated with the prevention of cardiovascular disease in menopausal and postmenopausal Australian women. Health Care Women Int. 27, 238–253. 10.1080/07399330500506543 16524854

[B4] Anderson D. Seib C. Rasmussen L. (2014). Can physical activity prevent physical and cognitive decline in postmenopausal women? A systematic review of the literature. Maturitas 79, 14–33. 10.1016/j.maturitas.2014.06.010 25008420

[B5] Arigo D. (2015). Promoting physical activity among women using wearable technology and online social connectivity: A feasibility study. Health Psychol. Behav. Med. 3, 391–409. 10.1080/21642850.2015.1118350

[B6] Arigo D. Brown M. M. Pasko K. Ainsworth M. C. Travers L. Gupta A. A. . (2020a). Rationale and design of the Women's Health And Daily Experiences project: Protocol for an ecological momentary assessment study to identify real-time predictors of midlife women's physical activity. JMIR Res. Protocols 9, e19044. 10.2196/19044 33055065 PMC7596655

[B7] Arigo D. Jake-Schoffman D. E. Wolin K. Beckjord E. Hekler E. B. Pagoto S. L. (2019). The history and future of digital health in the field of behavioral medicine. J. Behav. Med. 42, 67–83. 10.1007/s10865-018-9966-z 30825090 PMC6644720

[B8] Arigo D. Mogle J. A. Brown M. M. Roberts S. R. Pasko K. Butryn M. L. . (2020b). Differences in accelerometer cut point methods among midlife women with cardiovascular risk markers. Menopause 27, 559–567. 10.1097/GME.0000000000001498 32049926 PMC7903971

[B9] Arigo D. Mogle J. A. Smyth J. M. (2021). Relations between social comparisons and physical activity among women in midlife with elevated risk for cardiovascular disease: An ecological momentary assessment study. J. Behav. Med. 44, 579–590. 10.1007/s10865-021-00229-7 33982214 PMC8115872

[B10] Arigo D. Schumacher L. M. Pinkasavage E. Butryn M. L. (2015). Addressing barriers to physical activity among women: A feasibility study using social networking-enabled technology. Digital Health 1,2055207615583564. 10.1177/2055207615583564 PMC5999060 29942539

[B11] Arksey H. O'Malley L. (2005). Scoping studies: towards a methodological framework. Int. J. Soc. Res. Methodol 8, 19–32. 10.1080/1364557032000119616

[B12] Asbury E. A. Chandrruangphen P. Collins P. (2006). The importance of continued exercise participation in quality of life and psychological well-being in previously inactive postmenopausal women: a pilot study. Menopause 13, 561–567. 10.1097/01.gme.0000196812.96128.e8 16837877

[B13] Bandura A. (1982). Self-efficacy mechanism in human agency. Am. Psychol. 37, 122–147. 10.1037/0003-066X.37.2.122

[B14] Bandura A. (1998). Health promotion from the perspective of social cognitive theory. Psychol. Health 13, 623–649. 10.1080/0887044980840742231295924

[B15] Berkman L. F. Soh Y. (2017). Social determinants of health at older ages: The long arm of early and middle adulthood. Perspect. Biol. Med. 60, 595–606. 10.1353/pbm.2017.0045 29576566

[B16] Berry R. Kassavou A. Sutton S. (2021). Does self-monitoring diet and physical activity behaviors using digital technology support adults with obesity or overweight to lose weight? A systematic literature review with meta-analysis. Obesity Rev. 22, e13306. 10.1111/obr.13306 34192411

[B17] Blair S. N. (2009). Physical inactivity: The biggest public health problem of the 21st century. Br. J. Sport Med. 43, 1–2. Available online at: https://bjsm.bmj.com/content/bjsports/43/1/1.full.pdf?casa_token=JJhanI0CDygAAAAA:FAQnP2-1Sewshx_lRqNpPJX9K1j5liWVe~M8taWjr4K-ZrDeQYZ91qjkCJHX4mWYNHOIo3Pd_pHTV 19136507

[B18] Butryn M. L. Arigo D. Raggio G. A. Colasanti M. Forman E. M. (2016). Enhancing physical activity promotion in midlife women with technology-based self-monitoring and social connectivity: A pilot study. J. Health Psychol. 21, 1548–55. 10.1177/1359105314558895 25488937

[B19] Butryn M. L. Forman E. Hoffman K. Shaw J. Juarascio A. (2011). A pilot study of acceptance and commitment therapy for promotion of physical activity. J. Phys. Activity Health 8, 516–522. 10.1123/jpah.8.4.516 21597124

[B20] Butryn M. L. Godfrey K. M. Call C. C. Forman E. M. Zhang F. Volpe S. L. (2021). Promotion of physical activity during weight loss maintenance: A randomized controlled trial. Health Psychol. 40, 178. 10.1037/hea0001043 33630639 PMC8341135

[B21] Butryn M. L. Kerrigan S. Arigo D. Raggio G. Forman E. M. (2018). Pilot test of an acceptance-based behavioral intervention to promote physical activity during weight loss maintenance. Behav. Med. 44, 77–87. 10.1080/08964289.2016.1170663 27100874

[B22] Carels R. A. Darby L. A. Cacciapaglia H. M. Douglass O. M. (2004). Reducing cardiovascular risk factors in postmenopausal women through a lifestyle change intervention. J. Women's Health 13, 412–426. 10.1089/154099904323087105 15186658

[B23] Carmack Taylor C. L. Kulik J. Badr H. Smith M. Basen-Engquist K. Penedo F. . (2007). A social comparison theory analysis of group composition and efficacy of cancer support group programs. Soc. Sci. Med. 65, 262–73. 10.1016/j.socscimed.2007.03.024 17448580

[B24] Caspersen C. J. Pereira M. A. Curran K. M. (2000). Changes in physical activity patterns in the United States, by sex and cross-sectional age. Med. Sci. Sports Exer. 32, 1601–1609. 10.1097/00005768-200009000-00013 10994912

[B25] Conroy M. B. Sward K. L. Spadaro K. C. Tudorascu D. Karpov I. Jones B. L. . (2015). Effectiveness of a physical activity and weight loss intervention for middle-aged women: healthy bodies, healthy hearts randomized trial. J. General Inter. Med. 30, 207–213. 10.1007/s11606-014-3077-5 25391601 PMC4314485

[B26] Cooke R. Jones A. (2017). Recruiting adult participants to physical activity intervention studies using sport: a systematic review. BMJ Open Sport Exerc. Med. 3, 231. 10.1136/bmjsem-2017-000231 28761714 PMC5530105

[B27] Costanzo C. Walker S. N. Yates B. C. McCabe B. Berg K. (2006). Physical activity counseling for older women. Western J. Nurs. Res. 28, 786–801. 10.1177/0193945906289495 17056774

[B28] Cussler E. C. Teixeira P. J. Going S. B. Houtkooper L. B. Metcalfe L. L. Blew R. M. . (2008). Maintenance of weight loss in overweight middle-aged women through the Internet. Obesity 16, 1052–1060. 10.1038/oby.2008.19 18309301

[B29] Ehlers D. K. Huberty J. L. de Vreede G. J. (2015). Can an evidence-based book club intervention delivered via a tablet computer improve physical activity in middle-aged women? Telemed. e-Health 21, 125–131. 10.1089/tmj.2013.0360 25526014

[B30] Epton T. Currie S. Armitage C. J. (2017). Unique effects of setting goals on behavior change: Systematic review and meta-analysis. J. Consult. Clin. Psychol. 85, 1182–1198. 10.1037/ccp0000260 29189034

[B31] Festinger L. (1954). A theory of social comparison processes. Human Relat. 7, 117–140. 10.1177/001872675400700202

[B32] Fitzgibbon M. L. Stolley M. R. Schiffer L. Sanchez-Johnsen L. A. Wells A. M. Dyer A. (2005). A combined breast health/weight loss intervention for Black women. Prevent. Med. 40, 373–383. 10.1016/j.ypmed.2004.06.018 15530590

[B33] Flannery C. Fredrix M. Olander E. K. McAuliffe F. M. Byrne M. Kearney P. M. (2019). Effectiveness of physical activity interventions for overweight and obesity during pregnancy: A systematic review of the content of behaviour change interventions. Int. J. Behav. Nutr. Phys. Activity 16, 1–20. 10.1186/s12966-019-0859-5 31675954 PMC6825353

[B34] Forman E. M. Butryn M. L. (2015). A new look at the science of weight control: How acceptance and commitment strategies can address the challenge of self-regulation. Appetite 84, 171–180. 10.1016/j.appet.2014.10.004 25445199 PMC4314333

[B35] Gabriel K. P. Conroy M. B. Schmid K. K. Storti K. L. High R. R. Underwood D. A. . (2011). The impact of weight and fat mass loss and increased physical Activity on physical function in overweight, postmenopausal Women: Results from the WOMAN study. Menopause. 18, 759–765. 10.1097/gme.0b013e31820acdcc 21705864 PMC3181090

[B36] Gaston M. H. Porter G. K. Thomas V. G. (2007). Prime Time Sister Circles: evaluating a gender-specific, culturally relevant health intervention to decrease major risk factors in mid-life African-American women. J. Nat. Med. Assoc. 99, 428–38. 17444433 PMC2569659

[B37] Gebretatyos H. Amanuel S. Ghirmai L. Gebreyohannes G. Tesfamariam E. H. (2020). Effect of health education on healthy nutrition and physical activity among female teachers aged 40–60 years in Asmara, Eritrea: a quasi experimental study. J. Nutr. Metabol. 2020, 5721053. 10.1155/2020/5721053 33062325 PMC7533026

[B38] Gilinsky A. S. Dale H. Robinson C. Hughes A. R. McInnes R. Lavallee D. (2015). Efficacy of physical activity interventions in post-natal populations: Systematic review, meta-analysis and content coding of behaviour change techniques. Health Psychol. Rev. 9, 244–263. 10.1080/17437199.2014.899059 26209211

[B39] Gill J. M. R. Cooper A. R. (2008). Physical activity and prevention of type 2 diabetes mellitus. Sports Med. 38, 807–824. 10.2165/00007256-200838100-00002 18803434

[B40] Hayashi T. Farrell M. A. Chaput L. A. Rocha D. A. Hernandez M. (2010). Lifestyle intervention, behavioral changes, and improvement in cardiovascular risk profiles in the California WISEWOMAN project. J. Women's Health 19, 1129–1138. 10.1089/jwh.2009.1631 20509780

[B41] Hayes S. C. Luoma J. B. Bond F. W. Masuda A. Lillis J. (2006). Acceptance and commitment therapy: Model, processes and outcomes. Behav. Res. Ther. 44, 1–25. 10.1016/j.brat.2005.06.006 16300724

[B42] Hollis J. L. Williams L. T. Morgan P. J. Collins C. E. (2015). The 40-Something Randomised Controlled Trial improved fruit intake and nutrient density of the diet in mid-age women. Nutr. Dietet. 72, 316–326. 10.1111/1747-0080.12215

[B43] Hollis J. L. Williams L. T. Young M. D. Pollard K. T. Collins C. E. Morgan P. J. (2014). Compliance to step count and vegetable serve recommendations mediates weight gain prevention in mid-age, premenopausal women. Findings of the 40-Something RCT. Appetite 83, 33–41. 10.1016/j.appet.2014.07.020 25062965

[B44] Hu F. B. Stampfer M. J. Colditz G. A. Ascherio A. Rexrode K. M. Willett W. C. . (2000). Physical activity and risk of stroke in women. J. Am. Med. Assoc. 283, 2961–2967. 10.1001/jama.283.22.2961 10865274

[B45] Im E. O. Chang S. J. Chee W. Chee E. (2012). Attitudes of women in midlife to web-based interventions for promoting physical activity. J. Telemed. Telecare 18, 419–422. 10.1258/jtt.2012.120514 23104771

[B46] Im E. O. Stuifbergen A. K. Walker L. (2010). A situation-specific theory of midlife women's attitudes toward physical activity (MAPA). Nurs. Outlook 58, 52–58. 10.1016/j.outlook.2009.07.001 20113755 PMC2830712

[B47] Infurna F. J. Gerstorf D. Lachman M. E. (2020). Midlife in the 2020s: Opportunities and challenges. Am. Psychol. 75, 470–485. 10.1037/amp0000591 32378943 PMC7347230

[B48] Jenkins F. Jenkins C. Gregoski M. J. Magwood G. S. (2017). Interventions promoting physical activity in African American women: An integrative review. J. Cardiov. Nurs. 32, 22–29. 10.1097/JCN.0000000000000298 26544170 PMC4860177

[B49] Joseph R. P. Ainsworth B. E. Hollingshead K. Todd M. Keller C. (2021). Results of a culturally tailored smartphone-delivered physical activity intervention among midlife African American women: Feasibility trial. JMIR mHealth uHealth. 9, e27383. 10.2196/27383 33885368 PMC8103296

[B50] Joseph R. P. Royse K. E. Benitez T. J. (2019). A systematic review of electronic and mobile health (e- and mHealth) physical activity interventions for African American and Hispanic women. J. Phys. Activ. Health. 16, 230–239. 10.1123/jpah.2018-0103 30782040

[B51] Karvinen S. Jergenson M. J. Hyvärinen M. Aukee P. Tammelin T. Sipil,ä S. . (2019). Menopausal status and physical activity are independently associated with cardiovascular risk factors of healthy middle-aged women: Cross-sectional and longitudinal evidence. Front. Endocrinol. 10, 589. 10.3389/fendo.2019.00589 31543865 PMC6729112

[B52] Keyserling T. C. Hodge C. D. S. Jilcott S. B. Johnston L. F. Garcia B. A. Gizlice Z. . (2008). Randomized trial of a clinic-based, community-supported, lifestyle intervention to improve physical activity and diet: the North Carolina enhanced WISEWOMAN project. Prev. Med. 46, 499–510. 10.1016/j.ypmed.2008.02.011 18394692

[B53] Khare M. M. Carpenter R. A. Huber R. Bates N. J. Cursio J. F. Balmer P. W. . (2012). Lifestyle intervention and cardiovascular risk reduction in the Illinois WISEWOMAN Program. J. Women's Health. 21, 294–301. 10.1089/jwh.2011.2926 22136298

[B54] Kim Y. Kang S. (2020). Effects of a weight control intervention based on the transtheoretical model on physical activity and psychological variables in middle-aged obese women. J. Women Aging. 33, 556–68. 10.1080/08952841.2020.1728183 32174245

[B55] Kim Y. Lee Y. M. Cho M. Lee H. (2019). Effect of a pedometer-based, 24-week walking intervention on depression and acculturative stress among migrant women workers. Int. J. Environ. Res. Public Health 16, 4385. 10.3390/ijerph16224385 31717608 PMC6888469

[B56] Koniak-Griffin D. Brecht M. L. Takayanagi S. Villegas J. Melendrez M. Balcázar H. (2015). A community health worker-led lifestyle behavior intervention for Latina (Hispanic) women: Feasibility and outcomes of a randomized controlled trial. Int. J. Nursing Stud. 52, 75–87. 10.1016/j.ijnurstu.2014.09.005 25307195 PMC4277872

[B57] Krebs P. Prochaska J. O. Rossi J. S. (2010). A meta-analysis of computer-tailored interventions for health behavior change. Prev. Med. 51, 214–21. 10.1016/j.ypmed.2010.06.004 20558196 PMC2939185

[B58] Kulik J. A. Mahler H. I. Moore P. J. (1996). Social comparison and affiliation under threat: Effects on recovery from major surgery. J. Person. Soc. Psychol. 71, 967–979. 10.1037/0022-3514.71.5.967 8939044

[B59] Kuller L. H. Gabriel K. K. P. Kinzel L. S. Underwood D. A. Conroy M. B. Chang Y. . (2012). The Women on the Move Through Activity and Nutrition (WOMAN) study: final 48-month results. Obesity 20, 636–643. 10.1038/oby.2011.80 21494228 PMC3623568

[B60] Lee W.-C. Ory M. G. (2013). The Engagement in Physical Activity for Middle-Aged and Older Adults with Multiple Chronic Conditions: Findings from a Community Health Assessment. J. Aging Res. 2013, e152868. 10.1155/2013/152868 24089637 PMC3780626

[B61] Lin C.-H. Chiang S.-L. Yates P. Lee M.-S. Hung Y.-J. Tzeng W.-C. . (2015). Moderate physical activity level as a protective factor against metabolic syndrome in middle-aged and older women. J. Clin. Nurs. 24, 1234–1245. 10.1111/jocn.12683 25257388

[B62] Long J. E. Ring C. Bosch J. A. Eves F. Drayson M. T. Calver R. . (2013). A life-style physical activity intervention and the antibody response to pneumococcal vaccination in women. Psychosom. Med. 75, 774–782. 10.1097/PSY.0b013e3182a0b664 23922400

[B63] Low V. Gebhart B. Reich C. (2015). Effects of a worksite program to improve the cardiovascular health of female health care workers. J. Cardiopul. Rehabilit. Preven. 35, 342–7. 10.1097/HCR.0000000000000116 25853229

[B64] Ludman E. Simon G. E. Ichikawa L. E. Operskalski B. H. Arterburn D. Linde J. A. . (2009). Does depression reduce the effectiveness of behavioral weight loss treatment? Behav. Med. 35, 126–134. 10.1080/08964280903334527 19933059

[B65] Lynch B. M. Neilson H. K. Friedenreich C. M. (2011). Physical activity and breast cancer prevention. Recent Results Cancer Res. 186, 13–42. 10.1007/978-3-642-04231-7_2 21113759

[B66] Matthews K. A. Crawford S. L. Chae C. U. Everson-Rose S. A. Sowers M. F. Sternfeld B. . (2009). Are changes in cardiovascular disease risk factors in midlife women due to chronological aging or to the menopausal transition? J. Am. Coll. Cardiol 54, 2366–2373. /j.jacc.2009 10.1016/j.jacc.2009.10.009 20082925 PMC2856606

[B67] McGuire A. M. Seib C. Porter-Steele J. Anderson D. J. (2019). The association between Web-based or face-to-face lifestyle interventions on the perceived benefits and barriers to exercise in midlife women: Three-Arm Equivalency Study. J. Med. Internet Res. 21, e10963. 10.2196/10963 31436162 PMC6724500

[B68] Michie S. Ashford S. Sniehotta F. F. Dombrowski S. U. Bishop A. French D. P. (2011). A refined taxonomy of behaviour change techniques to help people change their physical activity and healthy eating behaviours: the CALO-RE taxonomy. Psychol. Health 26, 1479–1498. 10.1080/08870446.2010.540664 21678185

[B69] Michie S. Richardson M. Johnston M. Abraham C. Francis J. Hardeman W. . (2013). The behavior change technique taxonomy (v1) of 93 hierarchically clustered techniques: building an international consensus for the reporting of behavior change interventions. Ann. Behav. Med. 46, 81–95. 10.1007/s12160-013-9486-6 23512568

[B70] Mirzaei E. Azar F. E. F. Ziapour A. Azadi N. A. Qorbani M. Safari O. . (2020). The Impact of Educational Intervention Based on Theory of Planned Behavior for Promoting Physical Activity Among Middle-Aged Women Referring to Karaj (Iran) Health Centers. Int. Quart. Commun. Health Educ. 41, 419–426. 10.1177/0272684X20972849 33183170

[B71] Moore S. C. Lee I.-M. Weiderpass E. Campbell P. T. Sampson J. N. Kitahara C. M. . (2016). Association of leisure-time physical activity with risk of 26 types of cancer in 1.44 million adults. JAMA Internal Med. 176, 816–825. 10.1001/jamainternmed.2016.1548 27183032 PMC5812009

[B72] Murray J. M. Brennan S. F. French D. P. Patterson C. C. Kee F. Hunter R. F. (2017). Effectiveness of physical activity interventions in achieving behaviour change maintenance in young and middle aged adults: A systematic review and meta-analysis. Soc. Sci. Med. 192, 125–133. 10.1016/j.socscimed.2017.09.021 28965003

[B73] Napolitano M. A. Whiteley J. A. Papandonatos G. Dutton G. Farrell N. C. Albrecht A. . (2006). Outcomes from the women's wellness project: A community-focused physical activity trial for women. Prev. Med. 43, 447–453. 10.1016/j.ypmed.2006.06.011 16919322

[B74] National Center for Health Statistics (2019). Health, United States, 2019: Table 25. Hyattsville, MD. 2021. Available online at: https://www.cdc.gov/nchs/hus/contents2019.htm (accessed January 03, 2022).

[B75] Nazari L. N. Reisi M. Tahmasebi R. Javadzade H. (2020). The effect of web-based educational intervention on physical activity-related energy expenditure among middle-aged women with overweight and obesity: an application of social cognitive theory. Obesity Med. 18, 100181. 10.1016/j.obmed.2020.100181

[B76] Ortí E. S. Donaghy M. (2004). A Cognitive–behavioural intervention to increase adherence of adult women exercisers. Adv. Physiother. 6, 84–92. 10.1080/14038190410020115

[B77] Perez A. Fleury J. Keller C. (2010). Review of intervention studies promoting physical activity in Hispanic women. Western J. Nurs. Res. 32, 341–362. 10.1177/0193945909351300 20040732 PMC3152463

[B78] Peterson J. A. Yates B. C. Atwood J. R. Hertzog M. (2005). Effects of a physical activity intervention for women. Western J. Nurs. Res. 27, 93–110. 10.1177/0193945904270912 15659587

[B79] Pop M. Salzberg S. L. (2015). Use and mis-use of supplementary material in science publications. BMC Bioinform. 16, 1–4. 10.1186/s12859-015-0668-z 26525146 PMC4630891

[B80] Powell K. E. Paluch A. E. Blair S. N. (2011). Physical activity for health: What kind? How much? How intense? On top of what? Ann. Rev. Public Health 32, 349–365. 10.1146/annurev-publhealth-031210-101151 21128761

[B81] Prestwich A. Sniehotta F. F. Whittington C. Dombrowski S. U. Rogers L. Michie S. (2014). Does theory influence the effectiveness of health behavior interventions? Meta-analysis. Health Psychol. 33, 465–474. 10.1037/a0032853 23730717

[B82] Ribeiro M. A. Martins M. A. Carvalho C. R. (2014). Interventions to increase physical activity in middle-age women at the workplace: a randomized controlled trial. Med. Sci. Sports Exer. 46, 1008–15. 10.1249/MSS.0000000000000190 24126967

[B83] Samuel-Hodge C. D. Garcia B. A. Johnston L. F. Gizlice Z. Ni A. Cai J. . (2013). Translation of a behavioral weight loss intervention for mid-life, low-income women in local health departments. Obesity 21, 1764–1773. 10.1002/oby.20317 23408464

[B84] Samuel-Hodge C. D. Johnston L. F. Gizlice Z. Garcia B. A. Lindsley S. C. Bramble K. P. . (2009). Randomized trial of a behavioral weight loss intervention for low-income women: the Weight Wise program. Obesity. 17, 1891–1899. 10.1038/oby.2009.128 19407810

[B85] Scarinci I. C. Moore A. Wynn-Wallace T. Cherrington A. Fouad M. Li Y. (2014). A community-based, culturally relevant intervention to promote healthy eating and physical activity among middle-aged African American women in rural Alabama: Findings from a group randomized controlled trial. Prev. Med. 69, 13–20. 10.1016/j.ypmed.2014.08.016 25152504 PMC4469991

[B86] Shariati M. Astaneh H. P. Khedmat L. Khatami F. (2021). Promoting sustainable physical activity among middle-aged Iranian women: a conceptual model-based interventional study. BMC Women's Health 21, 1–7. 10.1186/s12905-020-01152-w 33388051 PMC7777291

[B87] Sharpe P. A. Burroughs E. L. Granner M. L. Wilcox S. Hutto B. E. Bryant C. A. . (2010). Impact of a community-based prevention marketing intervention to promote physical activity among middle-aged women. Health Educ. Behav. 37, 403–423. 10.1177/1090198109341929 19875639

[B88] Sherifali D. Nerenberg K. A. Wilson S. Semeniuk K. Ali M. U. Redman L. M. . (2017). The effectiveness of eHealth technologies on weight management in pregnant and postpartum women: Systematic review and meta-analysis. J. Med. Internet Res. 19, e8006. 10.2196/jmir.8006 29030327 PMC5660296

[B89] Shirazi K. K. Wallace L. M. Niknami S. Hidarnia A. Torkaman G. Gilchrist M. . (2007). A home-based, transtheoretical change model designed strength training intervention to increase exercise to prevent osteoporosis in Iranian women aged 40–65 years: a randomized controlled trial. Health Educ. Res. 22, 305–317. 10.1093/her/cyl067 16928779

[B90] Silva M. N. Marques M. M. Teixeira P. J. (2014). Testing theory in practice: The example of self-determination theory-based interventions. Eur. Health Psychol. 16, 171–180.

[B91] Simkin-Silverman L. R. Wing R. R. Boraz M. A. Kuller L. H. (2003). Lifestyle intervention can prevent weight gain during menopause: results from a 5-year randomized clinical trial. Ann. Behav. Med. 26, 212–220. 10.1207/S15324796ABM2603_06 14644697

[B92] Thomas V. G. Gaston M. H. Porter G. K. Anderson A. (2016). Prime time sister Circles^®^ II: evaluating a culturally relevant intervention to decrease psychological and physical risk factors for chronic disease in mid-life African American women. J. Nat. Med. Assoc. 108, 6–18. 10.1016/j.jnma.2015.12.001 26928483

[B93] Tovar M. Walker J. L. Rew L. (2018). Factors associated with physical Activity in Latina women: A systematic review. Western J. Nurs. Res. 40, 270–297. 10.1177/0193945916681004 27920349

[B94] Tremblay M. S. Aubert S. Barnes J. D. Saunders T. J. Carson V. Latimer-Cheung A. E. . (2017). Sedentary behavior research network (SBRN)–terminology consensus project process and outcome. Int. J. Behav. Nutr. Phys. Activ. 14, 1–17. 10.1186/s12966-017-0525-8 28599680 PMC5466781

[B95] Tricco A. C. Lillie E. Zarin W. O'Brien K. K. Colquhoun H. Levac D. . (2018). PRISMA extension for scoping reviews (PRISMA-ScR): checklist and explanation. Ann. Internal Med. 169, 467–473. 10.7326/M18-0850 30178033

[B96] Troiano R. P. Berrigan D. Dodd K. W. Masse L. C. Tilert T. McDowell M. (2008). Physical activity in the United States measured by accelerometer. Med. Sci. Sports Exer. 40, 181. 10.1249/mss.0b013e31815a51b3 18091006

[B97] Vrazel J. Saunders R. P. Wilcox S. (2008). An overview and proposed framework of social-environmental influences on the physical-activity behavior of women. Am. J. Health Prom. 23, 2–12. 10.4278/ajhp.06070999 18785368

[B98] Waters L. A. Galichet B. Owen N. Eakin E. (2011). Who participates in physical activity intervention trials? J. Phys. Activity Health 8, 85–103. 10.1123/jpah.8.1.85 21297189

[B99] Watson S. L. Weeks B. K. Weis L. J. Harding A. T. Horan S. A. Beck B. R. (2018). High-intensity resistance and impact training improves bone mineral density and physical function in postmenopausal women with osteopenia and osteoporosis: The LIFTMOR randomized controlled trial. J. Bone Mineral Res. 33, 211–220. 10.1002/jbmr.3284 28975661

[B100] White B. M. Rochell J. K. Warren J. R. (2020). Promoting cardiovascular health for African American women: An integrative review of interventions. J. Women's Health 29, 952–970. 10.1089/jwh.2018.7580 31502905

[B101] Wilbur J. Chandler P. Miller A. M. Davis G. C. Aaronson L. S. Mayo K. (2001). Measuring adherence to a women's walking program. Western J. Nurs. Res. 23, 8–32. 10.1177/01939450122044934 11216027

[B102] Wilbur J. McDevitt J. H. Wang E. Dancy B. L. Miller A. M. Briller J. . (2008). Outcomes of a home-based walking program for African-American women. Am. J. Health Prom. 22, 307–317. 10.4278/ajhp.22.5.307 18517090

[B103] Wilbur J. Miller A. M. Buchholz S. W. Fogg L. F. Braun L. T. Halloway S. . (2017). African-American women's long-term maintenance of physical activity following a randomized controlled trial. Am. J. Health Behav. 41, 484–496. 10.5993/AJHB.41.4.13 28601108

[B104] Wilbur J. Miller A. M. Fogg L. McDevitt J. Castro C. M. Schoeny M. E. . (2016). Randomized clinical trial of the women's lifestyle physical activity program for African-American women: 24-and 48-week outcomes. Am. J. Health Prom. 30, 335–345. 10.1177/0890117116646342 27404642

[B105] Wilbur J. Vassalo A. Chandler P. McDevitt J. Miller A. M. (2005). Midlife women's adherence to home-based walking during maintenance. Nurs. Res. 54, 33–40. 10.1097/00006199-200501000-00005 15695937

[B106] Wills T. A. (1985). “Supportive functions of interpersonal relationships,” in Social Support and Health. (pp. 61–82). Academic Press. Available online at: https://psycnet.apa.org/record/1985-97489-004 (accessed January 03, 2022).

[B107] Zenk S. N. Wilbur J. Wang E. McDevitt J. Oh A. Block R. . (2009). Neighborhood environment and adherence to a walking intervention in African American women. Health Educ. Behav. 36, 167–181. 10.1177/1090198108321249 18669878 PMC2726823

