# Supplementary material for: A scoping review of behavior change techniques used to promote physical activity among women in midlife
Source: Front Psychol. 2022 Sep 21;13:855749. doi: 10.3389/fpsyg.2022.855749 (PMC9534296; doi:10.3389/fpsyg.2022.855749)
Supplement: Supplementary file 1 [file Table_1.pdf]

Supplementary Table A. *Descriptive information for articles describing a physical activity intervention for women in midlife (k = 51).*

Authors and Year	Sample Size	Sample Characteristics	Primary or Secondary Outcomes	Length of Active Intervention	Format(s)	Control or Comparison Condition (Yes/No)	Delivery Modality or Modalities
Agomo et al. (2015)	20	Women 40-64 years old, at or below 250% of the federal poverty level, uninsured or underinsured	Primary	4 weeks	Group	No	In person
Anderson et al. (2006)	90	Women 45-60 year old, from high/low socio-economic and rural/metropolitan areas	Primary	12 weeks	Individual	Yes	Hybrid (in person and web)
Asbury et al. (2006)	18	Women 50-65 years old, healthy, postmenopausal (absence of menstruation for at least 1 y)	Primary	6 weeks	Individual	Yes	In person
Butryn et al. (2016)	26	Women 40-65 years old, <150 minutes of MVPA per week, with internet access	Primary	6 months	Group	No	Hybrid (in person and web)
Carels et al. (2004)	44	Women who were postmenopausal (no menstruation for at least 12 months), obese (BMI 30+ kg/m2), sedentary (not participating in a program of physical conditioning two or more times per week for at least 20 minutes per session, nonsmokers, no significant medical history	Secondary	6 months	Group	Yes	In person
Conroy et al. (2015)	98	Women 45-65 years old, BMI>25, <60 minutes of PA/week, patient in primary care clinic	Primary	12 weeks	Group	Yes	In person
Costanzo et al. (2006)	46	Women 50-65 years old, readiness for physical activity (Physical Activity Readiness Questionnaire or written clearance from a physician)	Primary	12 weeks	Individual	Yes	In person

Supplementary Table A. *Descriptive information for articles describing a physical activity intervention for women in midlife (k = 51).*

Authors and Year	Sample Size	Sample Characteristics	Primary or Secondary Outcomes	Length of Active Intervention	Format(s)	Control or Comparison Condition (Yes/No)	Delivery Modality or Modalities
Cussler et al. (2008)	135	Women 40-55 years old, BMI 25-38 kg/m2, perimenopausal (no definition provided)	Secondary	16 months	Group and individual	Yes	In person, then web (for maintenance)
Ehlers et al. (2015)	20	Women 30-64 years old, not meeting PA guidelines	Primary	12 weeks	Group and individual	Yes	in person or web (depended on condition)
Fitzgibbon et al. (2005)	64	Women 35-65 years old, BMI>25 kg/m2, self-identification as African American or Black	Primary	20 weeks	Group	Yes	In person
Gabriel et al. (2011)	508	Women 52-62 years old, postmenopausal (no definition provided), BMI 25-39.9 kg/m2, waist circumference ≥ 80 cm, blood pressure ≤ 140/90 mmHg, low density lipoprotein cholesterol level 100–160 mg/dL	Secondary	36 months	Group	Yes	In person
Gaston et al. (2007)	106	Women >35 years old, self-identification as African American or Black	Primary	10 weeks	Group	Yes	In person
Gebretatyos et al. (2020)	99	Women 40-60 years old, working in elementary, junior, or secondary schools	Primary	3 months	Group	No	In person
Hayashi et al. (2010)	645	Women 40-64 years old, <200% of the federal poverty level, under- or uninsured	Primary	6 months	Individual	Yes	In person
Hollis et al. (2014)	54	Women 44–50 years old, non-obese (BMI<30 kg/m2), pre-menopausal, healthy	Secondary	12 months	Group	Yes	Hybrid (in person, mailing, print materials)
Hollis et al. (2015)	54	Women 44–50 years old, premenopausal (no definition provided), BMI 18.5-29.9 kg/m2	Secondary	12 months	Group	Yes	Hybrid (in person and written materials)

Supplementary Table A. *Descriptive information for articles describing a physical activity intervention for women in midlife (k = 51).*

Authors and Year	Sample Size	Sample Characteristics	Primary or Secondary Outcomes	Length of Active Intervention	Format(s)	Control or Comparison Condition (Yes/No)	Delivery Modality or Modalities
Keyserling et al. (2008)	236	Women 40-64 years old, income at or below 200% of the federal poverty level	Primary	12 months	Group and individual	Yes	Hybrid (in person, phone, mail, and electronic resources)
Khare et al. (2012)	833	Women 40-64 years old, income <200% of the federal poverty level, under- or uninsured	Primary	12 weeks	Individual	Yes	Hybrid (in person and written materials)
Kim (2020)	41	Women 45-54 years old, in perimenopause, menopause, or postmenopause, sedentary (not defined)	Primary	16 weeks	Individual	No	In person
Kim & Kang (2020)	33	BMI>25, not currently dieting, midlife not defined (sample mean age=48)	Primary	12 weeks	Individual	No	In person
Kim et al. (2019)	84	Women 40-65 years old, not engaging in regular exercise, self-identification as a Korean migrant worker	Primary	24 weeks	Individual	Yes	Text message
Koniak-Griffin et al. (2015)	210	Women 35-64 years old, BMI>25 kg/m2, otherwise healthy, self-identified as Latina	Primary	6 months	Group and individual	Yes	Hybrid (in person and phone)
Kuller et al. (2012)	508	Women 52-62 years old, BMI 25-39.9 kg/m2, waist circumference ≥ 80 cm, blood pressure ≤ 140/90 mmHg, low density lipoprotein cholesterol level 100–160 mg/dL	Primary	48 months	Group	Yes	In person

Supplementary Table A. *Descriptive information for articles describing a physical activity intervention for women in midlife (k = 51).*

**Authors and Year**	**Sample Size**	**Sample Characteristics**	**Primary or Secondary Outcomes**	**Length of Active Intervention**	**Format(s)**	**Control or Comparison Condition (Yes/No)**	**Delivery Modality or Modalities**
Long et al. (2013)	89	Women 35-65 years old, insufficiently active (participated in 30 minutes of moderate-intensity exercise on 5 days of the week)	Primary	16 weeks	Individual	Yes	Hybrid (in person + phone, email, or text)
Low et al. (2015)	57	Women 40-65 years old, ≥1 risk factors for CVD (overweight, high stress level, lack of physical activity, or smoking)	Primary	6 months	Group and individual	Yes	Hybrid (in person, phone, and email)
Ludman et al. (2010)	190	Women 40-65 years old, self-reported BMI>30 kg/m2, current depressive disorder (PHQ-9)	Secondary	12 months	Group	No	In person
McGuire et al. (2018)	157	Women 40-65 years old (additional details provided elsewhere)	Secondary	12 weeks	Individual	Yes	Hybrid (in person and web)
Mirzaei et al. (2020)	140	Women 30-50 years old	Primary	2 months	Individual	Yes	In person
Napolitano et al. (2006)	280	Inactive (<90 min of purposeful moderate-intensity physical activity per week. e.g., walking or <60min of vigorous intensity activity, midlife not defined (sample mean age=47)	Primary	4-6 months	Individual	Yes	Print materials sent via mail
Nazari et al. (2020)	91	Women 30-59 years old, BMI>24.99 kg/m2, at least 12 years of education	Primary	3 months	Individual	Yes	In person
Newman et al. (2009)	318	Women 45-64 years old, postmenopausal (defined by age), waist circumference >80 cm, BMI 25-39.9 kg/m2, low density lipoprotein level 100–160 mg/dL	Secondary	18 months	Group	Yes	In person

Supplementary Table A. *Descriptive information for articles describing a physical activity intervention for women in midlife (k = 51).*

Authors and Year	Sample Size	Sample Characteristics	Primary or Secondary Outcomes	Length of Active Intervention	Format(s)	Control or Comparison Condition (Yes/No)	Delivery Modality or Modalities
Orti & Donaghy (2004)	27	Women 49-69 years old, sedentary (fewer than 3 sessions of 20 min per week of exercise), normal bone mineral density or osteopenia, rural residence	Primary	4 weeks	Group and individual	Yes	In person
Peterson et al. (2005)	42	Women 35-65 years old, currently inactive (<30 minutes of moderate-intensity activity daily)	Primary	12 weeks	Group and individual	Yes	in person or phone (depended on condition)
Ribeiro et al. (2014)	195	Physically inactive (<30 min of PA in leisure time), BMI<40 kg/m2, healthy, midlife not defined (sample of women 40-50 years old)	Primary	3 months	Group or individual (depended on condition)	Yes	in person
Samuel-Hodge et al. (2009)	143	Women 40–64 years old, income ≤200% of the federal poverty level, uninsured or under-insured, BMI 25-45 kg/m2	Primary	16 weeks	Group or individual (depended on condition)	Yes	In person or print (control)
Samuel-Hodge et al. (2013)	126	Women 40–64 years old, income ≤200% of the federal poverty level, BMI 25-45 kg/m3	Primary	16 weeks	Group	Yes	In person
Scarinci et al. (2014)	309	Women 45-65 years old, self-identification as African American	Primary	5 weeks	Group and individual	Yes	Hybrid (in person and phone)
Sedlak et al. (2005)	124	Women 50-65 years old, postmenopausal (no menstrual cycle in the past 12 months)	Primary	Self-administered	Print materials via mail	Yes	Phone and mail
Shariati et al. (2021)	80	Women 30-59 years old, inactive (<90 min mod-intensity PA/week), healthy	Primary	3 months	individual	Yes	Hybrid (in person and virtual meetings)

Supplementary Table A. *Descriptive information for articles describing a physical activity intervention for women in midlife (k = 51).*

**Authors and Year**	**Sample Size**	**Sample Characteristics**	**Primary or Secondary Outcomes**	**Length of Active Intervention**	**Format(s)**	**Control or Comparison Condition (Yes/No)**	**Delivery Modality or Modalities**
Sharpe et al. (2010)	217	Women 35-54 years old, insufficiently active (<30 minutes of moderate-intensity activity on 5 days per week or <20 minutes per day of vigorous- intensity activity on 3 days per week)	Primary	24 weeks	Group and individual	Yes	in person, phone, emailed and print mailed materials
Shirazi et al. (2007)	116	Women 40-65 years old, at least 5th grade education	Secondary	12 weeks	Group and individual	Yes	In person
Simkin-Silverman et al. (2003)	509	Women 44-50 years old, premenopausal, not taking hormone replacement therapy, BMI 20-34 kg/m2, fasting total cholesterol 140-260 mg/dl, fasting low density lipoproteins 80-160 mg/dl, fasting glucose level <140 mg/dl, diastolic blood pressure <95 mm Hg	Secondary	54 months	Group (all), individual (subset)	Yes	In person
Thomas et al. (2016)	922	Self-identification as African-American, midlife not defined (sample mean age=55)	Primary	12 weeks	Group	Yes	In person
Wilbur et al. (2001)	156	Women 45-65 years old, healthy, employed, self-identification as African American or Caucasian, sedentary (not participating in either physical conditioning or a sport for 20 minutes or more per session, two or more times per week during the preceding 6 months)	Primary	24 weeks	Individual	No	In person

Supplementary Table A. *Descriptive information for articles describing a physical activity intervention for women in midlife (k = 51).*

**Authors and Year**	**Sample Size**	**Sample Characteristics**	**Primary or Secondary Outcomes**	**Length of Active Intervention**	**Format(s)**	**Control or Comparison Condition (Yes/No)**	**Delivery Modality or Modalities**
Wilbur et al. (2005)	90	Women 45-65 years old, healthy, employed, self-identification as African American or Caucasian, sedentary (not participating in either physical conditioning or a sport for 20 minutes or more per session, two or more times per week during the preceding 6 months)	Primary	6 months	Individual	No	In person
Wilbur et al. (2008)	281	Women 45-65 years old, healthy, employed, self-identification as African American, sedentary (not participating in either physical conditioning or a sport for 20 minutes or more per session, two or more times per week during the preceding 6 months)	Primary	48 weeks	Group and individual	Yes	Hybrid (in person and phone)
Wilbur et al. (2016)	288	Women 45-65 years old, self-identification as African American	Primary	48 weeks	Group and individual	No	Hybrid (in person and phone)
Wilbur et al. (2017)	288	Women 45-65 years old, healthy, employed, self-identification as African American, sedentary (not participating in either physical conditioning or a sport for 20 minutes or more per session, two or more times per week during the preceding 6 months)	Secondary	24 weeks	Group and individual	Yes	Hybrid (in person and phone)

Supplementary Table A. *Descriptive information for articles describing a physical activity intervention for women in midlife (k = 51).*

Authors and Year	Sample Size	Sample Characteristics	Primary or Secondary Outcomes	Length of Active Intervention	Format(s)	Control or Comparison Condition (Yes/No)	Delivery Modality or Modalities
Wildman et al. (2004)	353	Women 44-50 years old, premenopausal (<3 months of amenorrhea in the preceding 6 months), high-normal ranges of diastolic BP, BMI, fasting glucose, and cholesterol levels	Secondary	20 weeks	Group and individual	Yes	In person
Xi et al. (2017)	55	Women 40-55 years old, mild to moderate perimenopausal syndrome, Kupperman Menopause Index (KMI) score 15-35	Primary	12 weeks	Individual	Yes	Hybrid (in person, phone, and text)
Zenk et al. (2009)	252	Women ages 40-65, sedentary (no participation in regular moderate or vigorous exercise for 30 minutes two or more times a week in the preceding 6 months), self-identification as African-American, urban or suburban residence, no signs of CVD	Secondary	12 months	Individual	Yes	Hybrid (in person and phone)

Note: PA = physical activity; BMI = body mass index.

Supplementary Table B. *Theories, behavior change techniques, and methods of activating behavior change techniques in physical activity interventions for women in midlife (k = 51).*

**Authors and Year**	**Theoretical Grounding**	**Goal Setting (1.1, 1.3)**	**Self-Monitoring (2.3, 2.4)**	**Feedback (2.2)**	**Planning/ Problem-Solving (1.2, 1.4)**	**Social Support (3.1, 3.2, 3.3)**	**Social Comparison (6.2)**	**Behavioral Modeling (6.1)**	**Other BCTs**
Agomo et al. (2015)	Social Cognitive Theory	Topic of discussion and activities during group sessions	N/A	N/A	Planning for achieving small behavior changes during group discussions	Discussion topic in group sessions	N/A	N/A	N/A
Anderson et al. (2006)	Bandura's Self-Efficacy model (Bandura, 1977)	Individual goal-setting session with nurse	Paper log	N/A	Planning (received weekly exercise planner in written materials)	Noted that support was available from staff but no detail provided	N/A	N/A	N/A
Asbury et al. (2006)	Not identified	N/A	Heart rate monitor, paper log	N/A		N/A	N/A	N/A	N/A
Butryn et al. (2016)	Not identified	Topic of discussion and activities during group sessions	Fitbit Flex	Intervention leaders provided feedback on progress	Planning in group discussions and activities	Facilitation of support (e.g., sharing feedback and tips) in group session, posts to community board between sessions via Fitbit platform	Data sharing between group members with leaderboard visible	N/A	Stimulus control in group discussions and activities (12.3)

Supplementary Table B. *Theories, behavior change techniques, and methods of activating behavior change techniques in physical activity interventions for women in midlife (k = 51).*

**Authors and Year**	**Theoretical Grounding**	**Goal Setting (1.1, 1.3)**	**Self-Monitoring (2.3, 2.4)**	**Feedback (2.2)**	**Planning/ Problem-Solving (1.2, 1.4)**	**Social Support (3.1, 3.2, 3.3)**	**Social Comparison (6.2)**	**Behavioral Modeling (6.1)**	**Other BCTs**
Carels et al. (2004)	Baumeister's Self-Control Theory	Topic of discussion and activities during group sessions	Caltrac accelerometer and paper log	N/A	N/A	Noted emphasis on relationships but no detail provided	N/A	N/A	N/A
Conroy et al. (2015)	Not identified	N/A	Pedometer and paper log	N/A	N/A	Discussion topic in group sessions	N/A	N/A	N/A
Costanzo et al. (2006)	Health Promotion Model, Social Cognitive Theory	Goals set/revised during behavioral counseling sessions:	Paper logs	N/A	Planning and problem-solving (to overcome PA barriers) discussed during individual sessions	Discussion topic in group sessions	N/A	Muscle-strengthening video with woman leading exercises	N/A

Supplementary Table B. *Theories, behavior change techniques, and methods of activating behavior change techniques in physical activity interventions for women in midlife (k = 51).*

Authors and Year	Theoretical Grounding	Goal Setting (1.1, 1.3)	Self-Monitoring (2.3, 2.4)	Feedback (2.2)	Planning/ Problem-Solving (1.2, 1.4)	Social Support (3.1, 3.2, 3.3)	Social Comparison (6.2)	Behavioral Modeling (6.1)	Other BCTs
Cussler et al. (2008)	Not identified	N/A	Pedometers and paper or electronic logs	Electronic report of weight loss progress available during web phase	Problem-solving (i.e., overcoming barriers to exercise) during group discussions	Participant-organized online support groups, monitored by research staff	N/A	N/A	
Ehlers et al. (2015)	Social Cognitive Theory	N/A	Pedometer and online log	Instant PA feedback (graphs) from online pedometer log	Planning presented via workbook and group discussions (in person or via internet discussion board)	Topic in e-book and group discussions	N/A	N/A	
Fitzgibbon et al. (2005)	Social Cognitive Theory	Topic of discussion and activities during group sessions	PA self-monitoring referenced but method not provided	N/A	Planning in group discussions	Discussion topic in group sessions	N/A	Indicated as included in group sessions but model not specified	Stimulus control (12.3) and reinforcement (many possible) in group discussions
Gabriel et al. (2011)	Not identified	N/A	N/A	N/A		N/A	N/A	N/A	N/A

Supplementary Table B. *Theories, behavior change techniques, and methods of activating behavior change techniques in physical activity interventions for women in midlife (k = 51).*

**Authors and Year**	**Theoretical Grounding**	**Goal Setting (1.1, 1.3)**	**Self-Monitoring (2.3, 2.4)**	**Feedback (2.2)**	**Planning/ Problem-Solving (1.2, 1.4)**	**Social Support (3.1, 3.2, 3.3)**	**Social Comparison (6.2)**	**Behavioral Modeling (6.1)**	**Other BCTs**
Gaston et al. (2007)	Integration of Social-Cognitive Theory, the Transtheoretical Model, and the Person-Extended Family-Neighborhood (PEN) Model	Topic of discussion and activities during group sessions	N/A	N/A	N/A	Noted support as part of rationale no detail provided	N/A	Indicated as included in the intervention but method and model not specified	Cognitive behavioral strategies (unspecified)
Gebretatyos et al. (2020)	Not identified	N/A	N/A	N/A	N/A	N/A	N/A	N/A	N/A
Hayashi et al. (2010)	Socioecological Model	Topic of discussion and activities during individual sessions	N/A	N/A	N/A	N/A	N/A	N/A	N/A
Hollis et al. (2014)	Social Cognitive Theory	N/A	N/A (only for assessments)	N/A	N/A	N/A	N/A	N/A	N/A
Hollis et al. (2015)	Not identified	N/A	Pedometer and paper log	N/A	N/A	N/A	N/A	N/A	Mailings included information about PA behavior change strategies (unspecified)

Supplementary Table B. *Theories, behavior change techniques, and methods of activating behavior change techniques in physical activity interventions for women in midlife (k = 51).*

**Authors and Year**	**Theoretical Grounding**	**Goal Setting (1.1, 1.3)**	**Self-Monitoring (2.3, 2.4)**	**Feedback (2.2)**	**Planning/ Problem-Solving (1.2, 1.4)**	**Social Support (3.1, 3.2, 3.3)**	**Social Comparison (6.2)**	**Behavioral Modeling (6.1)**	**Other BCTs**
Keyserling et al. (2008)	Not identified	Topic of discussion and activities during individual sessions	Pedometer	Intervention leaders provided individual feedback on progress	Planning and problem-solving in group and individual discussions	Noted that "sessions were designed to build social support" but no detail provided	N/A	In-session guided exercise activities (model not specified)	
Khare et al. (2012)	Not identified	N/A	N/A	N/A		N/A	N/A	N/A	N/A
Kim (2020)	Not identified	N/A	N/A	N/A		N/A	N/A	N/A	N/A
Kim & Kang (2020)	Transtheoretical Model	N/A	N/A	N/A		N/A	N/A	N/A	Reinforcement provided by intervention leaders (many possible)
Kim et al. (2019)	Not identified	N/A	Pedometer and paper log	N/A		N/A	N/A	N/A	N/A

Supplementary Table B. *Theories, behavior change techniques, and methods of activating behavior change techniques in physical activity interventions for women in midlife (k = 51).*

Authors and Year	Theoretical Grounding	Goal Setting (1.1, 1.3)	Self-Monitoring (2.3, 2.4)	Feedback (2.2)	Planning/ Problem-Solving (1.2, 1.4)	Social Support (3.1, 3.2, 3.3)	Social Comparison (6.2)	Behavioral Modeling (6.1)	Other BCTs
Koniak-Griffin et al. (2015)	Not identified	Topic of discussion and activities during individual sessions	Pedometer and paper log	N/A	Problem-solving guidance provided by intervention leader	Discussion topic in individual sessions	N/A	Instructor-led group exercises, videos, and role play during intervention sessions	N/A
Kuller et al. (2012)	Cognitive Behavioral Theory	N/A	N/A	N/A	N/A	N/A	N/A	N/A	N/A
Long et al. (2013)	Not identified	Topic of discussion during individual sessions and workbook activities	Pedometer	N/A	N/A	Topic in workbook	N/A	N/A	Intervention reviewed barriers to PA and relapse prevention (in which components these appeared not clear)
Low et al. (2015)	Not identified	Topic of discussion and activities during individual sessions	N/A	N/A	N/A	Noted group walks as a method of building support	N/A	N/A	N/A
Ludman et al. (2010)	Not identified	N/A	Paper log	N/A	Problem-solving in group discussions	N/A	N/A	N/A	N/A

Supplementary Table B. *Theories, behavior change techniques, and methods of activating behavior change techniques in physical activity interventions for women in midlife (k = 51).*

Authors and Year	Theoretical Grounding	Goal Setting (1.1, 1.3)	Self-Monitoring (2.3, 2.4)	Feedback (2.2)	Planning/ Problem-Solving (1.2, 1.4)	Social Support (3.1, 3.2, 3.3)	Social Comparison (6.2)	Behavioral Modeling (6.1)	Other BCTs
McGuire et al. (2018)	Social Cognitive Theory	Topic of discussion and activities during individual sessions	N/A	N/A	Weekly activity planning, barriers to PA reviewed in print and electronic materials, discussed in group	N/A	N/A	Photographic illustrations of strength exercises	N/A
Mirzaei et al. (2020)	Theory of Planned Behavior	N/A	N/A	Received feedback from research staff on PA, needs and obstacles to physical movement, and solutions (single session)	N/A	N/A	N/A	N/A	N/A
Napolitano et al. (2006)	Social Cognitive Theory, Trans-theoretical Model	Topic of workbook activities	N/A	Received tailored feedback from research team on "self-efficacy, barriers, benefits, social support, and goal setting" (baseline and months 1,3,6)	N/A	Topic in workbook	N/A	N/A	Booklet "matched to stage of change" (no details provided)

Supplementary Table B. *Theories, behavior change techniques, and methods of activating behavior change techniques in physical activity interventions for women in midlife (k = 51).*

Authors and Year	Theoretical Grounding	Goal Setting (1.1, 1.3)	Self-Monitoring (2.3, 2.4)	Feedback (2.2)	Planning/ Problem-Solving (1.2, 1.4)	Social Support (3.1, 3.2, 3.3)	Social Comparison (6.2)	Behavioral Modeling (6.1)	Other BCTs
Nazari et al. (2020)	Social Cognitive Theory (SCT)	Topic of chapter video	N/A	N/A	Chapter video about planning	Support persons invited to orientation session and topic in video chapter	Chapter video showed testimonial by peer who lost 17kg	Chapter video showed an exercise session led by professional sports coach leading exercises to be done at home	N/A
Newman et al. (2009)	Not identified	N/A	N/A	N/A	N/A	N/A	N/A	N/A	N/A
Orti & Donaghy (2004)	Transtheoretical Model	Topic of discussion and activities during group sessions	Paper log of heart rate and perceived exertion	Received feedback from research staff on achievement of target heart rate and extent of physiological arousal	N/A	N/A	N/A	N/A	Use of self-reward to reinforce PA and prevent relapse (10.9)

Supplementary Table B. *Theories, behavior change techniques, and methods of activating behavior change techniques in physical activity interventions for women in midlife (k = 51).*

Authors and Year	Theoretical Grounding	Goal Setting (1.1, 1.3)	Self-Monitoring (2.3, 2.4)	Feedback (2.2)	Planning/ Problem-Solving (1.2, 1.4)	Social Support (3.1, 3.2, 3.3)	Social Comparison (6.2)	Behavioral Modeling (6.1)	Other BCTs
Peterson et al. (2005)	Adaptation of Social Comparison Theory	Topic of discussion and activities during group sessions	Pedometer and paper log	Received positive feedback from intervention leader on fitness/goal progress during weekly group sessions	N/A	Sessions designed to facilitate social support from the leader and group members, including appraisal support (PA recommendations, safety, CVD prevention), belonging support (groups, spiritual messages, walking partners), tangible support (resources, facilities), and self-esteem support (feedback, self-monitoring, rewards)	Noted that intervention was based on social comparison theory but no detail provided	Noted that participants received a walking video and engaged in group PA, but modeling not specified	N/A
Ribeiro et al. (2014)	Not identified	N/A	Pedometer and paper log	N/A	Problem-solving in group discussions	Noted group walks as a method of building support	N/A	N/A	Use of self-rewards as reinforcement for PA in group discussions (10.9)

Supplementary Table B. *Theories, behavior change techniques, and methods of activating behavior change techniques in physical activity interventions for women in midlife (k = 51).*

**Authors and Year**	**Theoretical Grounding**	**Goal Setting (1.1, 1.3)**	**Self-Monitoring (2.3, 2.4)**	**Feedback (2.2)**	**Planning/ Problem-Solving (1.2, 1.4)**	**Social Support (3.1, 3.2, 3.3)**	**Social Comparison (6.2)**	**Behavioral Modeling (6.1)**	**Other BCTs**
Samuel-Hodge et al. (2009)	Cognitive-Behavioral Theory	Topic of discussion and activities during group or individual sessions	Paper log	Received tailored feedback on progress from research staff (weeks 6, 11, 16)	Problem-solving in group discussions	N/A	N/A	PA demonstrations in group or individual sessions (model not specified)	Relapse prevention in group discussions (code not clear)
Samuel-Hodge et al. (2013)	Not identified	Topic of discussion and activities during group sessions	Paper log	N/A	N/A	N/A	N/A	PA demonstrations in group sessions (model not specified)	N/A
Scarinci et al. (2014)	Social Cognitive Theory, Transtheoretical Model	Topic of discussion and activities during group and individual sessions	N/A	Intervention leaders provided individual feedback on progress	Problem-solving in activities and group and individual discussions	N/A	N/A	N/A	N/A
Sedlak et al. (2005)	Revised Health Belief Model (RHBM - Health Belief Model, Self-Efficacy)	N/A	N/A	N/A	N/A	N/A	N/A	N/A	N/A

Supplementary Table B. *Theories, behavior change techniques, and methods of activating behavior change techniques in physical activity interventions for women in midlife (k = 51).*

**Authors and Year**	**Theoretical Grounding**	**Goal Setting (1.1, 1.3)**	**Self-Monitoring (2.3, 2.4)**	**Feedback (2.2)**	**Planning/ Problem-Solving (1.2, 1.4)**	**Social Support (3.1, 3.2, 3.3)**	**Social Comparison (6.2)**	**Behavioral Modeling (6.1)**	**Other BCTs**
Shariati et al. (2021)	Health Belief Model (HBM)	Topic of audiobook component	N/A	N/A	N/A	N/A	N/A	Tutorial videos showing exercise movements	N/A
Sharpe et al. (2010)	Social Marketing Framework, Social Cognitive Theory	Topic of handouts received via mail	Paper log	N/A	Problem-solving, time management as topics in written materials	Topic in workbook and noted group walks as a method of building support	N/A	N/A	Relapse prevention as a topic in written materials (code unclear)
Shirazi et al. (2007)	Transtheoretical Model (TTM)	N/A	N/A	Intervention leaders provided feedback on progress	N/A	Discussion topic in group and individual sessions	N/A	N/A	Counter conditioning (14.7), reinforcement management (many possible), and stimulus control (12.3) discussed in group

Supplementary Table B. *Theories, behavior change techniques, and methods of activating behavior change techniques in physical activity interventions for women in midlife (k = 51).*

**Authors and Year**	**Theoretical Grounding**	**Goal Setting (1.1, 1.3)**	**Self-Monitoring (2.3, 2.4)**	**Feedback (2.2)**	**Planning/ Problem-Solving (1.2, 1.4)**	**Social Support (3.1, 3.2, 3.3)**	**Social Comparison (6.2)**	**Behavioral Modeling (6.1)**	**Other BCTs**
Simkin-Silverman et al. (2003)	Cognitive-Behavioral Theory	N/A	Paper log	Intervention leaders provided written feedback on progress	N/A	N/A	N/A	N/A	N/A
Thomas et al. (2016)	Integration of Social-Cognitive Theory, the Transtheoretical Model, and the Person-Extended Family-Neighborhood (PEN) Model	N/A	N/A	N/A	N/A	Intervention described as using a "supportive group format" but no detail provided	N/A	N/A	Cognitive behavioral strategies (unspecified)
Wilbur et al. (2001)	Transtheoretcial Model, Interaction Model of Health Behavior	N/A	Paper log	N/A	Problem-solving individual discussions	N/A	N/A	N/A	N/A
Wilbur et al. (2005)	Interaction Model of Client Health Behavior	N/A	Paper log	Graphs were available to provide feedback on intensity of exercise	Overcoming barriers in individual discussions	Emotional support and reinforcement was given in the form of feedback on progress	N/A	N/A	N/A

Supplementary Table B. *Theories, behavior change techniques, and methods of activating behavior change techniques in physical activity interventions for women in midlife (k = 51).*

**Authors and Year**	**Theoretical Grounding**	**Goal Setting (1.1, 1.3)**	**Self-Monitoring (2.3, 2.4)**	**Feedback (2.2)**	**Planning/ Problem-Solving (1.2, 1.4)**	**Social Support (3.1, 3.2, 3.3)**	**Social Comparison (6.2)**	**Behavioral Modeling (6.1)**	**Other BCTs**
Wilbur et al. (2008)	Interaction Model of Client Health Behavior, Social-Cognitive Theory	Topic of discussion and activities during individual sessions	Paper log	Intervention leader provided stage-matched supportive feedback, based on information reported to the automated telephone system	Problem-solving in group and individual discussions	Noted emphasis on facilitating support during intervention interactions, no other details provided	N/A	Group sessions began with videos featuring role models from the targeted communities discussing the workshop topic	N/A
Wilbur et al. (2016)	Social Cognitive Theory	Topic of discussion and activities during group sessions	Lifecorder EX accelerometer (data entered into telephone system)	Lay health educator provided feedback on steps reported to the telephone system	Problem-solving and time management in group and individual discussions	N/A	N/A	Group sessions began with videos featuring role models from the targeted communities discussing the workshop topic	N/A
Wilbur et al. (2017)	Social Cognitive Theory	Topic of discussion and activities during group and individual sessions	Accelerometer (unspecified, data entered into telephone system)	N/A	Problem-solving and time management in group and individual discussions	Discussion topic in group and individual sessions	N/A	Behavioral rehearsal through role modeling (model not specified)	N/A

Supplementary Table B. *Theories, behavior change techniques, and methods of activating behavior change techniques in physical activity interventions for women in midlife (k = 51).*

**Authors and Year**	**Theoretical Grounding**	**Goal Setting (1.1, 1.3)**	**Self-Monitoring (2.3, 2.4)**	**Feedback (2.2)**	**Planning/ Problem-Solving (1.2, 1.4)**	**Social Support (3.1, 3.2, 3.3)**	**Social Comparison (6.2)**	**Behavioral Modeling (6.1)**	**Other BCTs**
Wildman et al. (2004)	Not identified	N/A	N/A	N/A	N/A	N/A	N/A	N/A	N/A
Xi et al. (2017)	Not identified	N/A	N/A	N/A	N/A		N/A	N/A	N/A
Zenk et al. (2009)	Not identified	N/A	Heart rate monitor, paper log	N/A	Planning in individual discussions	Intervention leaders provided tailored, supportive telephone calls	N/A	N/A	N/A

Note: PA = physical activity, BCT = behavior change technique; numeric codes refer to Michie and colleagues' (2013) BCT classification system.
